# Two New Alkaloids and a Triterpenoid Glycoside from *Rosa roxburghii* with Antioxidant and Enzyme Inhibitory Activities

**DOI:** 10.3390/antiox15060680

**Published:** 2026-05-28

**Authors:** Lang Zhou, Yin-Ju Zhang, Wen-Xia Dai, Fa-Ju Chen, Xiong Pan, Yu Wang, Li-Shou Yang, Qi-Ji Li, Xiao-Sheng Yang

**Affiliations:** 1 State Key Laboratory of Discovery and Utilization of Functional Components in Traditional Chinese Medicine, Guiyang 550014, China; 2 Natural Products Research Center of Guizhou Province, Guizhou Medical University, Guiyang 550014, China; 3 School of Pharmacy, Guizhou University of Traditional Chinese Medicine, Guiyang 550025, China

**Keywords:** *Rosa roxburghii* Tratt., alkaloids, antioxidant, *α*-glucosidase inhibition, cytotoxicity

## Abstract

*Rosa roxburghii* is a distinctive medicinal and edible plant resource. In an effort to uncover structurally novel secondary metabolites from this plant and explore their pharmacological potential, a phytochemical investigation of the fresh fruits of *R. roxburghii* led to the isolation of two new dihydroavicine alkaloids, roxburghcids D (**1**) and E (**2**), and a new triterpenoid glycoside, roxburghcid C (**6**), together with five known compounds (**3**–**5**, **7**–**8**). The structures of the new compounds were unambiguously elucidated through extensive spectroscopic analyses (including 1D and 2D NMR and HRESIMS), quantum chemical calculations, and single-crystal X-ray diffraction. In bioassay evaluations, compounds **2** and **3** exhibited significant ABTS radical scavenging activities, with IC_50_ values of 39.59 and 17.38 μM, respectively. Additionally, compounds **2**, **6**, and **8** exhibited modest α-glucosidase inhibitory activities, with inhibition rates of 33.13%, 38.61%, and 36.85%, respectively, at a concentration of 100 μM. Furthermore, compounds **2** and **4** showed significant cytotoxicity against the A549 human cancer cell lines, with IC_50_ values of 7.23 and 8.36 μM, respectively. This study represents the first report of alkaloids isolated from *R. roxburghii*, enriching its phytochemical profile and providing valuable chemotaxonomic insights for this species.

## 1. Introduction

Oxygen is the foundational electron acceptor for aerobic life; nevertheless, the partial reduction of molecular oxygen during cellular respiration and normal metabolism inevitably yields highly reactive intermediate metabolites, predominantly reactive oxygen species (ROS) and reactive nitrogen species (RNS) [1,2]. Under physiological conditions, low-to-moderate concentrations of ROS/RNS function as crucial secondary messengers, playing indispensable roles in cellular signal transduction, host immune defense, and the maintenance of intracellular homeostasis [3]. However, when the overproduction of these highly reactive molecules overwhelms the scavenging capacity of endogenous antioxidant defense systems—comprising enzymes such as superoxide dismutase (SOD) and catalase (CAT), alongside the glutathione (GSH) network—the pro-oxidant/antioxidant equilibrium is disrupted, culminating in “oxidative stress” [4,5]. Extensive clinical and epidemiological evidence has substantiated that prolonged oxidative stress is not only a primary driver of the biological aging process [6] but is also deeply implicated in the pathogenesis and progression of cardiovascular diseases, neurodegenerative disorders, metabolic syndromes, and multiple malignancies [7]. In recent years, plant-derived natural products have garnered considerable attention as promising interventional candidates due to their extraordinary antioxidant capacities. Bioactive phytochemicals, including gallic acid, resveratrol, lycopene, and β-carotene, as well as vitamins C and E, have demonstrated potent efficacy in mitigating oxidative stress and neutralizing radical species in vivo [8]. Consequently, the continuous exploration of targeted therapeutic strategies capable of efficiently scavenging excessive reactive molecules or upregulating intrinsic antioxidant defense mechanisms holds profound clinical significance and translational medical value for the prevention and management of oxidative stress-related pathologies.

*Rosa roxburghii* Tratt. (Rosaceae) is a distinctive medicinal and edible plant resource native to Guizhou Province, China [9]. *R. roxburghii* has been designated as one of the twelve key characteristic and advantageous industries in Guizhou Province and is protected as a geographical indication product. By the end of 2022, the cultivation area of *R. roxburghii* in Guizhou had reached approximately 140,000 hectares, with an annual fresh fruit yield of 300,000 tons and a total industrial output value exceeding 15 billion RMB [10]. A systematic elucidation of the phytochemical basis of *R. roxburghii* is therefore a critical prerequisite for the development and expansion of its novel functional applications. *R. roxburghii* is traditionally utilized to invigorate the spleen, promote digestion, and act as an astringent agent [11]. Prior phytochemical studies on *R. roxburghii* have led to the isolation of various secondary metabolites, including triterpenoids [12], flavonoids [13], and phenolics [14]. In parallel, pharmacological evaluations have revealed that extracts or constituents from this plant possess diverse bioactivities, such as antidepressant [15], sedative–hypnotic [16], antitumor [17], and anti-aging [18] effects.

Driven by the objective to isolate novel secondary metabolites from this plant and elucidate their biomedical relevance, in the present study, A phytochemical investigation of the fruits of *R. roxburghii* led to the isolation and characterization of five alkaloids and three triterpenoid glycosides, including two new dihydroavicine-type alkaloids and a new oleanane-type triterpenoid glycoside. The chemical structures and absolute configurations of the new compounds were unambiguously elucidated through a comprehensive combination of extensive spectroscopic analyses, quantum chemical calculations, and single-crystal X-ray diffraction. Furthermore, selected isolated compounds were evaluated for their antioxidant capacities, α-glucosidase inhibitory potentials, and cytotoxic activities.

## 2. Materials and Methods

### 2.1. General Instrumentation and Reagents

Optical rotation values were determined at 25 °C using an Anton Paar MCP 500 digital polarimeter (Anton Paar GmbH, Graz, Austria). UV and ECD spectral analyses were performed on a Shimadzu-2600 spectrophotometer (Shimadzu Corporation, Kyoto, Japan) and an Applied Photophysics Chirascan spectrometer (Applied Photophysics Ltd., Leatherhead, UK), respectively. Infrared (IR) scans were conducted using KBr pellets on a Shimadzu IRAffinity-1 unit (Shimadzu Corporation, Kyoto, Japan). For 1D and 2D NMR experiments, Bruker AVANCE III-600 instruments (Bruker Corporation, Rheinstetten, Germany) were utilized, referencing TMS as the internal standard. HRESIMS measurements were taken on a Thermo Fisher Scientific Q Exactive Focus mass spectrometer (Thermo Fisher Scientific, Waltham, MA, USA). Semi-preparative HPLC separations were executed using a Shimadzu LC-20A system (Shimadzu Corporation, Kyoto, Japan) fitted with an SPD-20A UV detector and a Shim-pack GIS C18 column (10 × 250 mm, 5 μm). Silica gel (200–300 mesh; Qingdao Marine Chemical Group Co., Ltd., Qingdao, China) was used as the stationary phase for column chromatography.

Deuterated chloroform and methanol were purchased from Tenglong Weibo Technology (Qingdao, Shandong, China). Na_2_CO_3_, sodium dihydrogen phosphate, and disodium hydrogen phosphate were obtained from Shanghai Titan Scientific (Shanghai, China). Industrial-grade EtOH, petroleum ether, EtOAc, DCM, and MeOH were sourced from Chengdu Jinshan Chemical Reagent Co., Ltd. (Chengdu, Sichuan, China), while HPLC-grade methanol was provided by TEDIA (Fairfield, OH, USA). Biological reagents, including DMEM (Thermo Fisher Scientific, Waltham, MA, USA), penicillin–streptomycin (Suzhou Xinsaimei Biotechnology Co., Ltd., Suzhou, Jiangsu, China), Trypsin–EDTA Solution A (Shanghai Xiaopeng Biotechnology Co., Ltd., Shanghai, China), and fetal bovine serum (Beijing Lanjieke Technology Co., Ltd., Beijing, China), were used as received. The α-glucosidase, pNPG, and ABTS and DPPH assay kits were acquired from Shanghai Yuanye Bio-Technology Co., Ltd. (Shanghai, China).

### 2.2. Plant Material

In September 2023, fruit samples of *Rosa roxburghii* Tratt. were obtained from Longli County, Guizhou Province, P. R. China. Dr. Jingzhong Chen (Guizhou University of Traditional Chinese Medicine) was responsible for authenticating the botanical material. The voucher specimen (No. GZLL20230903) was lodged at the Natural Products Research Center of Guizhou Province.

### 2.3. Extraction and Isolation

Fresh fruits of *Rosa roxburghii* (150 kg) were sliced and extracted three times with 95% EtOH under reflux. The solvent was removed in vacuo to yield a crude extract (11.68 kg), which was suspended in H_2_O (20 L) and partitioned sequentially with petroleum ether (PE) and EtOAc. This process afforded a PE fraction (Fr. A, 64.97 g) and an EtOAc fraction (Fr. B, 914.50 g).

Fraction A was subjected to chromatography over silica gel (200–300 mesh) using a gradient elution of PE–EtOAc (1:0 to 1:1, *v*/*v*). The eluates were pooled based on TLC analysis to give nine subfractions (A1–A9). Further fractionation of A7 via silica gel CC (PE–EtOAc, 10:1 to 4:1, *v*/*v*) yielded subfractions A7-1–A7-3. Subfraction A7-3 was purified by silica gel CC with isocratic elution using dichloromethane (DCM) to afford compounds **1** (60.3 mg) and **2** (10.5 mg).

Fraction B was separated by silica gel CC (DCM–MeOH, 1:0 to 1:1, *v*/*v*) to yield six fractions (B1–B6). Fraction B2 was subjected to silica gel CC (DCM–MeOH, 30:1 to 1:1, *v*/*v*) to give subfractions B2-1–B2-5. Subsequent chromatography of B2-3 (DCM–MeOH, 80:1 to 1:1, *v*/*v*) resulted in subfractions B2-3-1–B2-3-4. Subfraction B2-3-2 was purified by silica gel CC (DCM–MeOH, 50:1, *v*/*v*) followed by semipreparative HPLC (60% MeOH–H_2_O, 2 mL/min) to yield **3** (80.1 mg, *t*_R_ = 25 min). Similarly, purification of B2-3-4 via silica gel CC (DCM–MeOH, 80:1, *v*/*v*) afforded **4** (52.1 mg). Fraction B2-4 was fractionated over silica gel (DCM–MeOH, 50:1 to 1:1, *v*/*v*) to produce subfractions B2-4-1–B2-4-4. Subfraction B2-4-1 was passed through a Sephadex LH-20 column (DCM–MeOH, 1:1, *v*/*v*) to give B2-4-1-1 and B2-4-1-2. Fr. B2-4-1-2 was purified by semipreparative HPLC (70% MeOH–H_2_O, 2 mL/min) to yield **5** (13.0 mg, *t*_R_ = 26 min).

Fraction B5 was subjected to medium-pressure liquid chromatography (MPLC) over reversed-phase C18 silica gel, eluting with a gradient of MeOH/H_2_O (10:90 to 60:40, *v*/*v*) to afford six subfractions (Fr. B5-1 to B5-6). Fraction B5-3 was first chromatographed over a silica gel column eluted with a gradient of CH_2_Cl_2_/MeOH (10:1 to 0:1, *v*/*v*), followed by size-exclusion chromatography on Sephadex LH-20 (CH_2_Cl_2_/MeOH, 1:1, *v*/*v*) to yield three subfractions (Fr. B5-3-1 to B5-3-3). Subsequently, subfraction B5-3-1-1 was purified by silica gel column chromatography (CH_2_Cl_2_/MeOH, 10:1, *v*/*v*) to give compound **8** (10 mg). Subfraction B5-3-1-2 was separated over a silica gel column (CH_2_Cl_2_/MeOH, 6:1, *v*/*v*) to afford additional compound **7** (7 mg). Subfraction B5-3-1-3 was purified by semi-preparative HPLC (isocratic 60% MeOH/H_2_O, 2.0 mL/min) to yield compound **6** (9.7 mg, *t*_R_ = 85 min).

#### 2.3.1. Roxburghcid D (**1**)

White powder; ${\left[\alpha \right]}_{D}^{25}$ −0.51 (c 0.23, MeOH); UV (MeOH) λ_max_ (log ε): 203 (3.93), 229 (3.70), 280 (3.58), 320 (3.58) nm; ECD (MeOH) *λ* (Δε) 217 (−0.84), 261 (−0.26) nm; IR (KBr) υ_max_ 3385, 2960, 2902, 1469, 1228, 1033, 941, 850, 806 cm^–1^; for ^1^H and ^13^C NMR data, see Table 1; HR-ESI-MS *m*/*z* 378.1333 [M + H]^+^ (calcd for C_22_H_20_O_5_N, 378.1336).

#### 2.3.2. Roxburghcid E (**2**)

White powder; ${\left[\alpha \right]}_{D}^{25}$ −1.05 (c 0.13, MeOH); UV (MeOH) λ_max_ (log ε): 204 (3.89), 231 (4.22), 281 (4.06), 317 (3.87) nm; ECD (MeOH) *λ* (Δε) 230 (4.28), 281 (0.80) nm; IR (KBr) υ_max_ 3566, 1481, 1465, 1247, 1037, 937, 864 cm^–1^; for ^1^H and ^13^C NMR data, see Table 1; HR-ESI-MS *m*/*z* 378.1331 [M + H]^+^ (calcd for C_22_H_20_O_5_N, 378.1336).

#### 2.3.3. Aegelbine-A (**3**)

Yellow needle crystals. ^1^H NMR (600 MHz, CD_3_OD) *δ*: 7.90 (1H, d, *J* = 9.5 Hz, H-5), 7.73 (1H, d, *J* = 2.2 Hz, H-2), 7.26 (1H, s, H-4), 6.81 (1H, d, *J* = 2.2 Hz, H-3), 6.29 (1H, d, *J* = 9.5 Hz, H-6); ^13^C NMR (150 MHz, CD_3_OD) *δ*: 148.3 (C-2), 107.9 (C-3), 111.2 (C-4), 147.1 (C-5), 114.6 (C-6), 141.1 (C-7), 147.1 (C-8), 163.0 (C-9), 127.3 (C-10), 117.8 (C-11), 131.7 (C-12).

#### 2.3.4. Canthine-6-one (**4**)

Yellow needle crystals. ^1^H NMR (600 MHz, CDCl_3_) *δ*: 8.80 (1H, d, *J* = 5.0 Hz, H-2), 8.63 (1H, d, *J* = 8.2 Hz, H-8), 8.07 (1H, d, *J* = 7.7 Hz, H-11), 8.00 (1H, dd, *J* = 9.8, 1.0 Hz, H-4), 7.93 (1H, d, *J* = 4.9 Hz, H-1), 7.68 (1H, t, *J* = 7.8 Hz, H-9), 7.50 (1H, t, *J* = 7.6 Hz, H-10), 6.96 (1H, d, *J* = 9.7 Hz, H-5); ^13^C NMR (150 MHz, CDCl_3_) *δ*: 116.5 (C-1), 145.9 (C-2), 139.7 (C-4), 159.6 (C-6), 117.4 (C-8), 131.0 (C-9), 125.8 (C-10), 122.8 (C-11), 124.5 (C-12), 139.5 (C-13), 130.4 (C-14), 132.1 (C-15), 136.3 (C-16).

#### 2.3.5. (R)-5-(1-Hydroxyethyl)-canthine-6-one (**5**)

Yellow needle crystals. ^1^H NMR (600 MHz, CD_3_OD) *δ*: 8.77 (1H, d, *J* = 5.0 Hz, H-2), 8.58 (1H, d, *J* = 8.2 Hz, H-8), 8.21 (2H, m, H-4, H-11), 8.11 (1H, d, *J* = 5.0 Hz, H-1), 7.74 (1H, m, H-9), 7.57 (1H, t, *J* = 7.6 Hz, H-10), 5.20 (1H, m, H-17), 1.62 (3H, d, *J* = 6.5 Hz, H-18); ^13^C NMR (150 MHz, CD_3_OD) *δ*: 117.3 (C-1), 146.5 (C-2), 133.3 (C-4), 147.5 (C-5), 160.0 (C-6), 117.8 (C-8), 131.8 (C-9), 126.9 (C-10), 124.1 (C-11), 125.8 (C-12), 140.5 (C-13), 131.5 (C-14), 132.2 (C-15), 136.8 (C-16), 65.9 (C-17), 23.4 (C-18).

#### 2.3.6. Roxburghcid C (**6**)

Needle crystals; ${\left[\alpha \right]}_{D}^{25}$ −36.7 (c 0.15, MeOH); UV (MeOH) λ_max_ (log ε): 203 (3.42), 255 (3.75) nm; IR (KBr) υ_max_ 3360, 2926, 2868, 1749, 1556, 1454, 1446, 1377, 1247, 1035, 1006, 871, 785, 596, 540 cm^–1^, for ^1^H and ^13^C NMR data, see Table 2; HR-ESI-MS *m*/*z* 671.3749 [M + H]^+^ (calcd for C_36_H_56_O_10_Na, 671.3766).

#### 2.3.7. 2α,3β,19α,23-Tetrahydroxylurs-12-en-28-oic Acid-β-D-glucopyranosyl Ester (**7**)

^1^H NMR (CD_3_OD, 600 MHz) *δ*_H_: 5.30 (2H, d, *J* = 8.2 Hz, H-12, H-1′), 3.35–3.78 (5H, H2′–6′), 3.66 (1H, m, H-2), 3.48 (1H, d, *J* = 11.1 Hz, H-23b), 3.25 (1H, d, J = 11.1 Hz, H-23a), 2.50 (1H, s, H-18), 1.32 (3H, s, H-27), 1.19 (3H, s, H-29), 1.02 (3H, s, H-25), 0.91 (3H, d, *J* = 7.0 Hz, H-30), 0.76 (3H, s, H-26), 0.68 (3H, s, H-24). ^13^C NMR (CD_3_OD, 500 MHz) *δ*_C_: 48.0 (C-1), 69.7 (C-2), 78.3 (C-3), 44.1 (C-4), 48.5 (C-5), 19.2 (C-6), 33.5 (C-7), 40.9 (C-8), 48.2 (C-9), 39.0 (C-10), 24.8 (C-11), 129.5 (C-12), 139.8 (C-13), 42.8 (C-14), 29.6 (C-15), 26.5 (C-16), 48.3 (C-17), 55.0 (C-18), 73.6 (C-19), 42.9 (C-20), 27.2 (C-21), 38.3 (C-22), 66.3 (C-23), 13.9 (C-24), 17.7 (C-25), 17.6 (C-26), 24.7 (C-27), 178.5 (C-28), 27.1 (C-29), 16.6 (C-30), 95.8 (C-1′), 73.8 (C-2′), 78.6 (C-3′), 71.1 (C-4′), 78.2 (C-5′), 62.4 (C-6′).

#### 2.3.8. 2α,3α,19α-Trihydroxy-olean-12-en-28-oic Acid-β-D-glucopyranosyl Ester (**8**)

White powder. ^1^H NMR (600 MHz, CD_3_OD) *δ*: 5.35 (1H, d, *J* = 8.1 Hz, H-1′), 5.32 (1H, t, *J* = 3.7 Hz, H-12), 1.29 (3H, s, H-27), 0.97 (3H, s, H-23), 0.97 (3H, s, H-29), 0.93 (3H, s, H-26), 0.92 (3H, s, H-25), 0.85 (3H, s, H-30), 0.72 (3H, s, H-24). ^13^C NMR (150 MHz, CD_3_OD) *δ*: 42.4 (C-1), 67.1 (C-2), 80.1 (C-3), 39.5 (C-4), 49.8 (C-5), 19.3 (C-6), 33.8 (C-7), 41.1 (C-8), 49.4 (C-9), 39.5 (C-10), 24.9 (C-11), 124.9 (C-12), 144.4 (C-13), 42.7 (C-14), 29.5 (C-15), 28.4 (C-16), 47.1 (C-17), 45.1 (C-18), 82.4 (C-19), 36.0 (C-20), 29.4 (C-21), 33.2 (C-22), 29.2 (C-23), 22.4 (C-24), 16.9 (C-25), 17.8 (C-26), 25.2 (C-27), 178.6 (C-28), 28.6 (C-29), 25.1 (C-30), 95.8 (C-1′), 73.9 (C-2′), 78.7 (C-3′), 71.1 (C-4′), 78.3 (C-5′), 62.4 (C-6′).

#### 2.3.9. Crystal Data of Roxburghcid C (**6**)

C_36_H_56_O_10_, M = 648.80 g/mol, monoclinic, space group P21 (no. 4), *a* = 15.5231(4) Å, *b* = 6.41190(10) Å, *c* = 16.9954(4) Å, *α* = 90°, *β* = 91.272(2), *γ* = 90°, *V* = 1691.18(6) Å3, *Z* = 2, *T* = 100.01(10) K, μ (Cu Kα) = 0.747 mm^−1^, Dcalc = 1.274 g/cm^3^, 10,601 reflections measured (5.2° ≤ 2Θ ≤ 148.004°), 4867 unique (*R*_int_ = 0.0498, *R*_sigma_ = 0.0689) which were used in all calculations. The final *R*_1_ was 0.0603 (I > 2σ(I)), and w*R*_2_ was 0.1601 (all data). Flack parameter = 0.3 (3). CCDC number: 2539246.

**Table 1:** NMR data of compounds **1**–**2**.

Pos.	1 ^a^		2 ^a^	
	*δ*_C_	*δ*_H_ (*J* in Hz)	*δ*_C_	*δ*_H_ (*J* in Hz)
1	104.1	7.30, s	104.3	7.24, s
2	147.2	-	147.2	-
3	147.9	-	147.9	-
4	100.4	7.83, s	99.7	7.48, s
4a	126.2	-	126.0	-
4b	139.0	-	139.8	-
6	69.9	3.71, d (8.7)	69.6	3.49, d (9.1)
6a	125.6	-	128.1	-
7	109.4	6.98, s	110.5	6.81, s
8	146.6	-	146.2	-
9	147.5	-	147.1	-
10	103.4	7.50, s	103.0	7.43, s
10a	124.8	-	124.2	-
10b	123.3	-	123.7	-
11	119.9	7.77, d (8.6)	120.1	7.77, d (8.6)
12	123.6	7.53, d (8.6)	123.4	7.46, d (8.6)
12a	130.6	-	130.5	-
1′	66.1	3.07, m	67.3	2.98, m
2′	19.5	0.89, d (6.2)	21.5	1.09, d (6.1)
N-Me	42.4	2.61, s	42.6	2.51, s
OCH_2_O-2/3	101.2	6.14, dd (4.6, 1.0)	101.2	6.08, dd (12.7, 1.2)
OCH_2_O-8/9	101.0	6.07, dd (6.8, 1.2)	100.8	5.99, dd (12.7, 1.2)

^a^ Measured at 150 MHz for ^13^C NMR and 600 MHz for ^1^H NMR in DMSO.

**Table 2:** NMR data of compound **6**.

Pos.	6 ^a^		Pos.	6 ^a^	
	*δ*_C_	*δ*_H_ (*J* in Hz)		*δ*_C_	*δ*_H_ (*J* in Hz)
1	41.7	1.19, m 1.68, m	19	33.6	2.67, m
2	64.5	3.79, m	20	26.9	1.82, m
3	72.4	3.06, q (8.4, 7.7)	21	24.2	1.14, m 1.62, m
4	43.9	-	22	31.1	1.37, m 2.10, dd (13.6, 9.3)
5	47.8	1.29, d (12.7)	23	22.9	0.95, s
6	18.0	1.37, m 1.46, d (13.0)	24	63.4	3.25, dd (11.1, 4.6) 3.42, dd (11.2, 5.3)
7	32.3	1.19, m	25	19.2	0.88, s
8	40.2	-	26	16.2	0.76, s
9	54.0	1.95, s	27	20.9	0.88, s
10	37.3	-	28	175.3	-
11	125.3	6.39, m	29	16.1	0.92, d (7.0)
12	126.7	5.67, d (10.6)	30	19.2	0.81, d (6.6)
13	134.5	-	1′	94.6	5.30, d (8.2)
14	41.6	-	2′	72.7	3.57, m
15	24.6	0.92, m 1.62, m	3′	76.6	3.20, m
16	31.7	1.55, m 1.82, m	4′	69.8	3.06, q (8.4, 7.7)
17	46.2	-	5′	77.7	3.20, m
18	137.4	-	6′	61.0	3.42, dd (11.2, 5.3) 3.65, dd (11.4, 4.9)

^a^ Measured at 200 MHz for ^13^C NMR and 800 MHz for ^1^H NMR in DMSO.

### 2.4. NMR and ECD Calculations

Conformational analyses for compounds **1** and **2** were initiated using the Spartan14 software. The resulting conformers were geometrically optimized at the B3LYP/6-31G(d) level in the gas phase using the Gaussian 09 program package [19]. Stable conformers with relative energies within a specific window were selected for further calculations, and their room-temperature equilibrium populations were determined according to the Boltzmann distribution law. For NMR calculations, Gauge-Independent Atomic Orbital (GIAO) shielding tensors were calculated at the mPW1PW91/6-311G(d,p) level using the PCM solvation model (DMSO) [20]. The calculated ^13^C and ^1^H shielding constants were subjected to DP4^+^ probability analysis to determine the most likely configuration [21]. ECD calculations were performed using Time-Dependent Density Functional Theory (TDDFT) [22] at the wB97XD/6-311G(d,p) level in methanol with the PCM solvation model. The final ECD spectra were simulated by Boltzmann weighting of the dominant conformers using a Gaussian function with a bandwidth (sigma/gamma) of 0.3 eV.

### 2.5. α-Glucosidase Inhibitory and Antioxidant Assay

The *α*-glucosidase inhibition activity was evaluated following a previously described protocol [23]. Briefly, sample solutions (25 μL) at varying concentrations (100 μM) were mixed with *α*-glucosidase (50 μL, 0.2 U/mL) in 96-well plates and pre-incubated at 37 °C for 10 min. The reaction was initiated by the addition of 5 mM *p*-nitrophenyl-*α*-D-glucopyranoside (pNPG, 25 μL), and after incubation at 37 °C for 5 min, the reaction was quenched with 100 μL of 0.1 M Na_2_CO_3_. Absorbance was subsequently recorded at 405 nm using a microplate reader. All experiments were performed in triplicate and independently repeated three times.

The ABTS and DPPH radical scavenging capacities were assessed using commercial kits according to the manufacturer’s instructions. For the ABTS assay, a working solution was prepared by diluting the stock ABTS radical cation with anhydrous EtOH. A 10 μL aliquot of the sample (100, 50, 25, 12.5, and 6.25 μM) was mixed with 190 μL of the ABTS working solution and incubated at 25 °C for 6 min in the dark. Absorbance was measured at 734 nm. For the DPPH assay, 50 μL of the sample (100 μM) was combined with 225 μL of EtOH and 225 μL of DPPH solution. Following incubation at 25 °C for 30 min in the dark, a 300 μL aliquot was transferred to a 96-well plate, and absorbance was recorded at 517 nm [24]. All experiments were performed in triplicate and independently repeated three times.

ABTS/DPPH radical scavenging rate (%) = [1 − (A_1_ − A_2_)/A_0_] × 100%

In this equation, A_0_, A_1_, and A_2_ correspond to the absorbance values recorded for the blank control, the experimental sample, and the sample control.

### 2.6. Cytotoxicity Assay

The in vitro antiproliferative activities of compounds **1**–**6** against three human cancer cell lines (A549, HepG2, and MCF-7) were evaluated using a Cell Counting Kit-8 (CCK-8) assay. Cells were cultured in Dulbecco’s Modified Eagle Medium (DMEM) supplemented with 10% fetal bovine serum (FBS) at 37 °C in a humidified atmosphere of 5% CO_2_. Briefly, A549 cells were seeded into 96-well plates at a density of 5 × 10^3^ cells/well, while HepG2 and MCF-7 cells were seeded at 1 × 10^4^ cells/well. After a 24 h incubation to allow for cell adherence, the cells were treated with the tested compounds at a concentration of 20 μM. Following an additional 24 h incubation, CCK-8 reagent was added to each well. After a 2 h colorimetric reaction period, the absorbance was measured at 450 nm using a Multiskan FC microplate reader (Thermo Fisher Scientific, Waltham, MA, USA). Cisplatin was employed as a positive control [25]. All experiments were performed in triplicate and independently repeated three times.

### 2.7. Statistical Analysis

Statistical analyses were performed using SPSS version 27.0. Data are presented as the mean ± standard deviation (SD). Comparisons were conducted using one-way analysis of variance (ANOVA), followed by the Least Significant Difference (LSD) and Tukey’s post hoc tests. A *p*-value < 0.05 or <0.01 was considered statistically significant. All experiments were performed in triplicate and independently repeated three times.

## 3. Results

### 3.1. Identification of New Compounds

Roxburghcid D (**1**) was isolated as a white amorphous powder. Its molecular formula was determined to be C_22_H_19_O_5_N by HRESIMS (*m/z* 378.1333 [M + H]^+^; calcd for C_22_H_20_O_5_N, 378.1336), requiring 13 indices of hydrogen deficiency (IHDs). The IR spectrum displayed absorption bands characteristic of hydroxyl (3385 cm^−1^), benzene ring (1469 cm^−1^), and methylenedioxy (941 cm^−1^) functionalities. The ^1^H NMR data for **1** (Table 1) revealed six aromatic protons [*δ*_H_ 7.83 (1H, s, H-4), 7.77 (1H, d, *J* = 8.6 Hz, H-11), 7.53 (1H, d, *J* = 8.6 Hz, H-12), 7.50 (1H, s, H-10), 7.30 (1H, s, H-1), and 6.98 (1H, s, H-7)]; two methylenedioxy groups [*δ*_H_ 6.14 (2H, dd, *J* = 4.6, 1.0 Hz, H-OCH_2_O-2/3), and 6.07 (1H, dd, *J* = 6.8, 1.2 Hz, H-OCH_2_O-8/9)]; an oxygenated methine [*δ*_H_ 3.07 (1H, m, H-1′)]; and two methyl resonances [*δ*_H_ 0.89 (3H, d, *J* = 6.2 Hz, H-2′) and an N-methyl *δ*_H_ 2.61 (3H, s)]. The ^13^C NMR and HSQC spectra revealed 22 carbon resonances, including two methyls [*δ*_C_ (19.5, C-2′) and (42.4, N-Me)]; two methylenedioxy carbons [*δ*_C_ 101.2 and 101.0]; eight methine carbons [an N-bearing carbon *δ*_C_ (69.9, C-6), an oxygenated carbon *δ*_C_ (66.1, C-1′), six aromatic carbons (104.1, C-1), (100.4, C-4), (109.4, C-7), (103.4, C-10), (119.9, C-11), and (123.6, C-12)]; and ten quaternary aromatic carbons [*δ*_C_ (147.2, C-2), (147.9, C-3), (126.2, C-4a), (139.0, C-4b), (125.6, C-6a), (146.6, C-8), (147.5, C-9), (124.8, C-10a), (123.3, C-10b), and (130.6, C-12a)].

**Figure 1:**
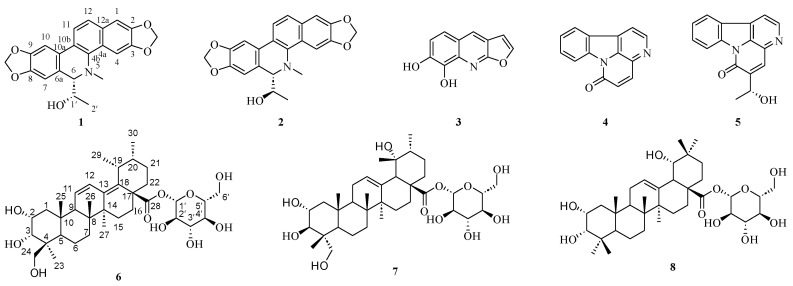
The structures of compounds **1**–**8**.

**Figure 2:**
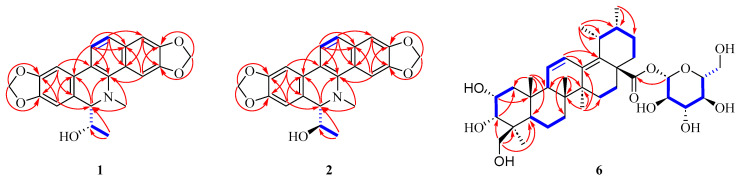
The key ^1^H-^1^H COSY (blue bold) and HMBC (red arrows) correlations of **1**–**2**, **6**.

**Figure 3:**
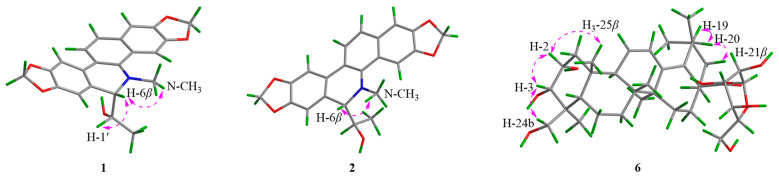
Key NOESY (purple arrows) correlations of **1**–**2**, **6**.

**Figure 4:**
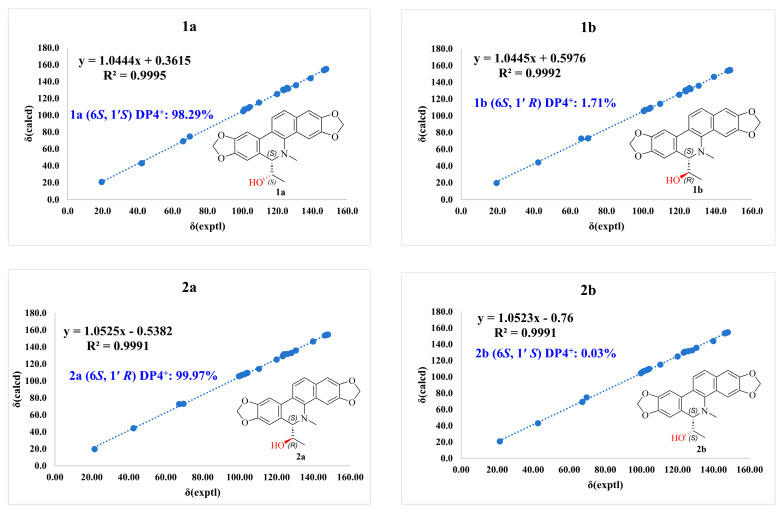
DP4^+^ probability analysis of compounds **1** and **2**.

**Figure 5:**
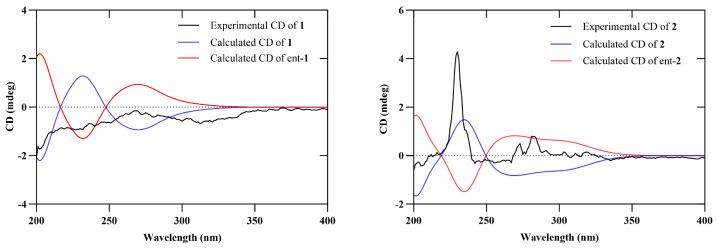
Experimental and calculated ECD spectra of compounds **1** and **2**.

Detailed analysis of the ^1^H and ^13^C NMR data suggested that compound **1** possesses a dihydroavicine alkaloid skeleton, structurally distinct from the known analogue 6-acetonyldihydroavicine [26] only by the substitution at C-6. Specifically, the acetonyl group in the analogue is replaced by a 1-hydroxyethyl moiety in **1** (Figure 1). This assignment was substantiated by key HMBC correlations (Figure 2) from H-2′ (*δ*_H_ 0.89) to C-1′ (*δ*_C_ 66.1) and C-6 (*δ*_C_ 69.9). The NOESY spectrum corroborated this relative configuration, with key correlations among *N*-CH_3_, H-6, and H-1′ indicating their co-facial *β*-orientation (Figure 3). By combining DP4^+^ probability analysis (Figure 4) and ECD calculations (Figure 5), the absolute configuration was determined to be 6*S*, 1′*S*, and named roxburghcid D.

Roxburghcid E (**2**) was obtained as a white amorphous powder. The molecular formula C_22_H_19_O_5_N was established by HR-ESI-MS ([M + H]^+^ 378.1331, C_22_H_20_O_5_N, calcd for 378.1336). The IR spectrum showed absorption bands for hydroxy (3566 cm^−1^), aromatic (1465 cm^−1^), and methylenedioxy (937 cm^−1^) functionalities. The ^1^H NMR and ^13^C NMR data of **2** (Table 1) closely resembled those of **1**, suggesting that they share the same planar constitution. However, minor differences in chemical shifts were observed, particularly around the chiral centers. Detailed analysis of 2D NMR correlations confirmed that **2** is the C-1′ epimer of **1**. The NOESY spectrum corroborated this relative configuration. Key correlations between the *N*-CH_3_ and H-6 protons indicated a co-facial relationship, establishing their *β*-orientations. In contrast, the lack of observable NOE cross-peaks between H-1′ and either the *N*-CH_3_/H-7 protons suggested they reside on opposite faces, permitting the assignment of H-1′ as *α*-oriented (Figure 3). The absolute configuration was established as (6*S*, 1′*R*) via DP4^+^ probability analysis (Figure 4) and ECD calculations (Figure 5).

Roxburghcid C (**6**) was isolated as colorless needles. Its molecular formula was established as C_36_H_56_O_10_ on the basis of the HRESIMS pseudomolecular ion peak at *m*/*z* 671.3749 [M + Na]^+^ (calcd for C_36_H_56_O_10_Na, 671.3766), requiring 9 IHDs. The ^1^H NMR data (Table 2) revealed the presence of six methyls [*δ*_H_ 0.95 (3H, s, H-23), 0.88 (3H, s, H-25), 0.76 (3H, s, H-26), 0.88 (3H, s, H-27), 0.92 (3H, d, *J* = 7.0 Hz, H-29), and 0.81 (3H, d, *J* = 6.6 Hz, H-30)], an oxygenated methylene [*δ*_H_ 3.25 (1H, dd, *J* = 11.1, 4.6 Hz, H-24a) and 3.42 (1H, dd, *J* = 11.2, 5.3 Hz, H-24b)], and two oxygenated methines [*δ*_H_ 3.79 (1H, m, H-2) and 3.06 (1H, q, *J* = 8.4, 7.7 Hz, H-3)]. Additionally, a set of proton signals indicative of a *β*-glucopyranosyl moiety was observed [*δ*_H_ 5.30 (1H, d, *J* = 8.2 Hz, H-1′), 3.57 (1H, m, H-2′), 3.20 (1H, m, H-3′), 3.06 (1H, q, *J* = 8.4, 7.7 Hz, H-4′), 3.20 (1H, m, H-5′), 3.42 (1H, dd, *J* = 11.2, 5.3 Hz, H-6′a), and 3.65 (1H, dd, *J* = 11.4, 4.9 Hz, H-6′b)]. The ^13^C NMR (Table 2) and HSQC spectra resolved 36 carbon resonances, which were classified into six methyls [*δ*_C_ 22.9 (C-23), 19.2 (C-25), 16.2 (C-26), 20.9 (C-27), 16.1 (C-29), and 19.2 (C-30)], nine methylenes [*δ*_C_ 41.7 (C-1), 18.0 (C-6), 32.3 (C-7), 24.6 (C-15), 31.7 (C-16), 24.2 (C-21), 31.1 (C-22), and two oxygenated carbons *δ*_C_ 63.4 (C-24) and 61.0 (C-6′)], thirteen methines [*δ*_C_ 47.8 (C-5), 54.0 (C-9), 33.6 (C-19), 26.9 (C-20), and seven oxygenated *δ*_C_ 64.5 (C-2), 72.4 (C-3), 94.6 (C-1′), 72.7 (C-2′), 76.6 (C-3′), 69.8 (C-4′), and 77.7 (C-5′)], and eight quaternary carbons [*δ*_C_ 43.9 (C-4), 40.2 (C-8), 37.3 (C-10), 41.6 (C-14), 46.2 (C-17), two olefinic carbons *δ*_C_ 134.5 (C-13) and 137.4 (C-18), and one ester carbonyl *δ*_C_ 175.3 (C-28)].

**Figure 6:**
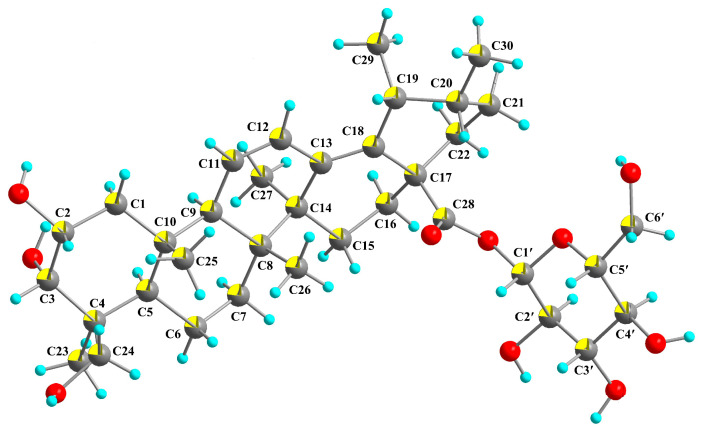
X-ray of compound **6**.

Analysis of the NMR data revealed that the structure of compound **6** is closely related to that of rubuside H [27], with the principal differences residing in their stereochemical configurations. In compound **6**, the hydroxyl groups at C-2 and C-3, as well as the methyl groups at C-4, C-19, and C-20, were assigned the *α*-orientation. This relative configuration was further corroborated by the NOESY spectrum. Key NOE correlations among H-24b/H-3/H-2/H_3_-25 indicated that these protons are co-facial, thereby establishing their *β*-orientation. Similarly, NOE cross-peaks observed among H-19/H-20/H-21*β* suggested a co-facial relationship, confirming the *β*-orientation of H-19 and H-20. Ultimately, a single-crystal X-ray diffraction analysis unambiguously established the absolute configuration of **6** as 2*R*, 3*S*, 4*S*, 5*R*, 8*R*, 9*R*, 10*S*, 14*S*, 17*S*, 19*R*, and 20*R* (CCDC No. 2539246) (Figure 6), and it was named roxburghcid C.

The known compounds were identified as aegelbine-A (**3**) [28], canthine-6-one (**4**) [29], (*R*)-5-(1-hydroxyethyl)-canthine-6-one (**5**) [30], 2*α*,3*β*,19*α*,23-tetrahydroxylurs-12-en-28-oic acid-β-D-glucopyranosyl ester (**7**) [31], and 2α,3α,19α-trihydroxy-olean-12-en-28-oic acid-β-D-glucopyranosyl ester (**8**) [32].

### 3.2. α-Glucosidase Inhibitory and Antioxidant Activities

The isolates were assessed for their *α*-glucosidase inhibitory and antioxidant properties in vitro. At a tested concentration of 100 μM, compounds **2**, **6**, and **8** showed moderate *α*-glucosidase inhibition (33.13%, 38.61%, and 36.85%, respectively). To assess variations in α-glucosidase inhibitory activity among the evaluated compounds, Tukey’s multiple-comparison test was applied. The results demonstrated comparable inhibitory effects among certain subsets of compounds; specifically, no statistically significant differences were observed between compound **8** and compounds **2** or **6**, nor between compound **5** and compounds **1**, **3**, or **4**.

**Figure 7:**
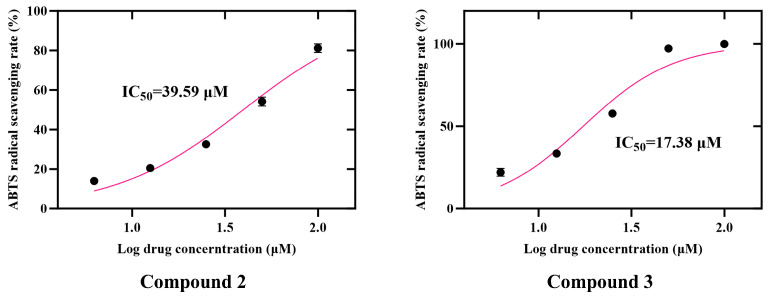
Concentration-dependent ABTS radical scavenging activity of compounds **2** and **3**.

In the ABTS radical scavenging assay, compounds **2** and **3** exhibited significant activities with respective inhibition rates of 81.18% and 99.97% at 100 μM. According to Tukey’s multiple-comparison test, compounds **2** and **3** demonstrated the highest ABTS radical scavenging potential, with a significant difference noted between the two. Furthermore, no significant differences in scavenging capacity were detected between compounds **1** and **6** or between compound **4** and compounds **5**, **6**, **7**, and **8**. Concentration-dependency evaluations further confirmed that compounds **2** and **3** exerted substantial dose-dependent effects across a concentration range of 6.25–100 μM, with calculated IC_50_ values of 39.59 μM and 17.38 μM, respectively (Figure 7).

Furthermore, compound **3** displayed moderate DPPH radical scavenging activity, achieving 25.94% at a concentration of 100 μM. According to Tukey’s post hoc analysis, compound **3** possessed the highest scavenging potential within the tested group, showing statistically significant differences compared to compounds **1**, **2**, and **4**–**8**. Moreover, no significant variations in scavenging efficacy were observed between compound **8** and compounds **1**, **2**, and **4**–**7**. All corresponding bioactivity results are detailed in Table 3.

### 3.3. In Vitro Cytotoxicity Assay of Compounds ***1***–***6***

**Figure 8:**
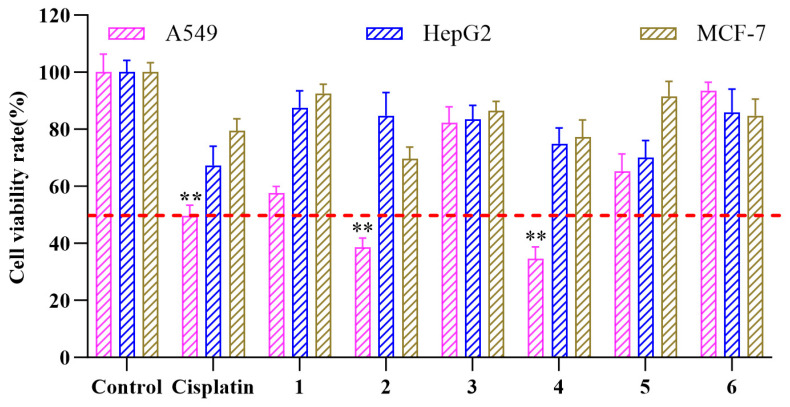
Cell viability of A549, HepG2, and MCF-7 cells following treatment with compounds **1**–**6** (20 µM). Data are expressed as mean ± SD (n = 3). ** *p* < 0.05 compared with the control.

**Figure 9:**
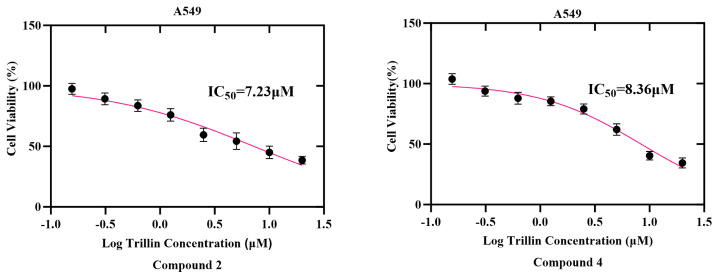
Dose-dependent inhibitory effects and IC_50_ values of compounds **2** and **4** against A549 cells.

The in vitro antiproliferative activities of compounds **1**–**6** were evaluated against a panel of three human cancer cell lines: A549 (lung), MCF-7 (breast), and HepG2 (liver). In an initial screening at 20 μM, compounds **2** and **4** exhibited potent growth inhibition against A549 cells, reducing cell viability to 38.60% and 34.50%, respectively (Figure 8). Subsequent concentration–response analyses determined the IC_50_ values of compounds **2** and **4** to be 7.23 and 8.36 μM, respectively (Figure 9).

## 4. Discussion

*R*. *roxburghii* has garnered considerable scientific attention due to its complex phytochemical profile and potent health-promoting properties. Renowned as a robust botanical source of dietary antioxidants, this species harbors a dynamic repository of enzymatic antioxidants—particularly superoxide dismutase (SOD)—and essential micronutrients. These components act synergistically with a diverse array of secondary metabolites, including triterpenoids, sesquiterpenoids, flavonoids, phenolic acids, organic acids, and polysaccharides. To date, phytochemical investigations have elucidated over 50 distinct compounds from *R. roxburghii*. Among these, pentacyclic triterpenoids emerge as the predominant class, comprising more than 33 identified structures characterized primarily as polyhydroxylated ursane- and oleanane-type scaffolds alongside their corresponding glycosides [12,13,33]. Additionally, approximately 15 sesquiterpenoids, largely monocyclic variants and their glycosidic derivatives, have been documented [33]. The application of high-resolution analytical techniques, such as LC-MS/MS, has further delineated the rich landscape of flavonoids and polyphenolics [34,35], solidifying the plant’s reputation as a premier source of natural antioxidants. Building upon this established phytochemical foundation, the present study provides a targeted exploration of the secondary metabolome in fresh *R. roxburghii* fruits. This endeavor yielded the successful isolation and structural elucidation of five alkaloids and three triterpenoid glycosides. Most notably, this study reports the first-ever discovery of alkaloidal constituents within *R. roxburghii*, specifically designated as roxburghcids D (**1**) and E (**2**). Furthermore, a new triterpenoid glycoside, roxburghcid C (**6**), was identified, featuring a relatively rare α-configuration of the methyl group at the C-29 position. The discovery of these structurally diverse and previously undescribed constituents substantially broadens the recognized phytochemical spectrum of *R. roxburghii*, providing novel molecular scaffolds for future pharmacological and antioxidant bioactivity investigations.

Previous studies have established that *R*. *roxburghii* extracts effectively mitigate oxidative stress by modulating endogenous cellular defense systems. For instance, in an H_2_O_2_-challenged HepG2 cell model, these extracts confer robust cytoprotection via a dual mechanism: directly attenuating the intracellular accumulation of reactive oxygen species (ROS) and indirectly upregulating critical antioxidant enzymes, such as superoxide dismutase (SOD) and glutathione peroxidase (GSH-Px), thereby restoring redox homeostasis [36]. Expanding upon these extract-level observations, the present study evaluates the intrinsic antioxidant potential of the specifically isolated constituents. Compounds **2** and **3** demonstrated significant ABTS radical scavenging activity, achieving inhibition rates between 81.18% and 99.97% at a screening concentration of 100 μM. Subsequent assays confirmed a distinct dose-dependent response across a concentration gradient (6.25–100 μM), yielding IC_50_ values of 39.59 and 17.38 μM, respectively. From a structure–activity relationship (SAR) perspective, the alkaloids exhibited superior ABTS radical scavenging activity compared to the triterpene glycosides. Notably, the two new alkaloids, compounds **1** and **2**, are epimers differing exclusively in the stereochemical configuration of the hydroxyl group at C-1. The isomer possessing the *S*-configuration (α-hydroxyl group) displayed a markedly lower ABTS scavenging capacity than its counterpart with the *R*-configuration (β-hydroxyl group). Classical reference antioxidants—such as resveratrol, vitamin E, and lycopene—typically exhibit potent in vitro scavenging capacities with IC_50_ values well below 10 μM [37]. In contrast, the individual compounds evaluated herein display relatively modest intrinsic efficacy (IC_50_ > 10 μM). Consequently, we postulate that rather than acting as singular, highly potent radical scavengers, these secondary metabolites function as integral co-contributors. They likely augment the overall antioxidant efficacy of *R. roxburghii* through additive or synergistic interactions within its complex phytochemical matrix.

Beyond its well-established antioxidant properties, *R*. *roxburghii* exhibits significant systemic efficacy in modulating glucolipid metabolism. In vitro studies have demonstrated that polyphenolic fractions enriched from *R. roxburghii* leaves exert robust inhibitory effects against *α*-glucosidase. Concurrently, in vivo evidence from diabetic animal models (e.g., db/db mice) reveals that these polyphenols promote hepatic glycogen synthesis and ameliorate insulin resistance via the upregulation of the PI3K/Akt signaling cascade. The integration of this systemic metabolic regulation with the localized inhibition of intestinal *α*-glucosidase establishes a potent, multi-target synergistic network for glycemic control [38]. Previous literature reports underscore that specific triterpenoids within *R. roxburghii*, such as rosamultic acid, roxbuterpene B, euscaphic acid, pomolic acid, and 2α,3α,24-trihydroxy-urs-12,18-diene-28-oic acid, possess potent *α*-glucosidase inhibitory capabilities (IC_50_ = 0.9–13.6 μM) [12]. Although the compounds screened in the present study exhibited relatively weak *α*-glucosidase inhibition, their combined intrinsic antioxidant capacities and metabolic regulatory potential suggest they play an indispensable role within the plant’s broader phytotherapeutic matrix. We propose that through dynamic synergy with highly active molecular scaffolds, these newly identified components contribute to a collective pharmacological interplay, thereby maintaining and amplifying the macroscopic efficacy of *R. roxburghii* against metabolic syndrome.

The dual role of oxidative stress in modulating tumor cell apoptosis remains a central paradigm in contemporary oncology research. In the context of hepatocellular carcinoma (HCC), previous studies have demonstrated that triterpenoid acid extracts from *R*. *roxburghii* (TAR) inhibit HepG2 cell proliferation and migration in a time- and dose-dependent manner. Mechanistically, these extracts induce a robust accumulation of intracellular reactive oxygen species (ROS). This supraphysiological ROS surge activates the c-Jun N-terminal kinase (JNK) signaling cascade, culminating in irreversible G2/M cell cycle arrest. Concurrently, the pronounced ROS generation triggers the dissipation of the mitochondrial membrane potential, facilitating the cytosolic translocation of cytochrome c. This process activates the caspase cascade, ultimately driving mitochondria-dependent apoptosis [39]. Building upon the chemical diversity elucidated earlier in this study, our preliminary in vitro screening identified promising antitumor properties among the newly isolated constituents. Notably, we report for the first time that roxburghcid E (**2**) and canthine-6-one (**4**)—alkaloids characterized from *R. roxburghii*—exhibit potent cytotoxicity against human non-small cell lung cancer A549 cells, yielding IC_50_ values of 7.23 and 8.36 μM, respectively. Structurally, alkaloids **2** and **4** exhibited significant inhibitory activity against A549 cells. Furthermore, the new alkaloids **1** and **2** differ solely in the stereochemical configuration of the hydroxyl group at the C-1′ position; notably, the epimer with the *α*-hydroxyl at C-1 displayed a substantially lower A549 inhibitory potency than that with the *β*-hydroxyl. These findings successfully correlate the structural diversity of the plant with its pharmacological efficacy. They not only significantly expand the known spectrum of bioactive compounds in *R. roxburghii* but also provide a preliminary basis for future lung cancer therapies targeting these specific alkaloids.

## 5. Conclusions

In this study, a phytochemical investigation of *R. roxburghii* fruits led to the isolation of two new dihydroavicine-type alkaloids, roxburghcids D (**1**) and E (**2**), and a new triterpenoid glycoside, roxburghcid C (**6**), alongside five known analogues. Structurally, compound **6** is notable as a rare ursane-type triterpenoid glycoside featuring an α-oriented C-29 methyl group. In in vitro biological evaluations, compounds **2** and **3** displayed significant ABTS radical scavenging capacities, while compounds **2**, **6**, and **8** exhibited moderate α-glucosidase inhibitory activities. Furthermore, compounds **2** and **4** demonstrated significant cytotoxicity against the A549 human lung cancer cell line. Importantly, this study constitutes the first report of alkaloids derived from *R. roxburghii*. These findings not only expand the known phytochemical space of this species but also provide a preliminary reference for its development and utilization as a medicinal and functional food resource.

## Figures and Tables

**Table 3:** *α*-glucosidase inhibitory and radical scavenging activities of compounds **1**–**8** (100 μM).

No.	*α*-Glucosidase Inhibition Rate (%)	Scavenging Rate of ABTS (%)	Scavenging Rate of DPPH (%)
**1**	4.30 ± 0.32 ^d^	11.55 ± 0.80 ^c^	10.43 ± 0.02 ^b^
**2**	33.13 ± 2.30 ^b^	81.18 ± 0.70 ^b^	10.14 ± 0.01 ^b^
**3**	−3.32 ± 1.85 ^e^	99.97 ± 0.06 ^a^	25.94 ± 0.01 ^a^
**4**	−1.58 ± 2.46 ^e^	5.88 ± 2.61 ^d^	3.11 ± 0.01 ^c^
**5**	0.56 ± 0.81 ^d^	2.36 ± 1.35 ^e^	9.92 ± 0.02 ^b^
**6**	38.61 ± 0.71 ^a^	9.13 ± 1.82 ^c^	4.05 ± 0.02 ^c^
**7**	16.61 ± 1.07 ^c^	6.38 ± 0.38 ^d^	10.18 ± 0.02 ^b^
**8**	36.85 ± 0.13 ^a^	4.20 ± 0.08 ^e^	7.02 ± 0.03 ^b^
Acarbose	99.87 ± 0.07	-	-
Trolox	-	9.97 ± 2.70	
Vitamin C	-	-	31.98 ± 1.51

One-way ANOVA followed by Tukey’s post hoc test. Data are expressed as mean ± SD (n = 3). Values sharing the same superscript letter are not significantly different (*p* > 0.05), whereas different letters indicate a significant difference (*p* < 0.05). Acarbose, Trolox, and Vitamin C were used as positive controls.

## Data Availability

The original contributions presented in this study are included in the article and Supplementary Materials. Further inquiries can be directed to the corresponding authors.

## Supplementary Materials

The following supporting information can be downloaded at: https://www.mdpi.com/article/10.3390/antiox15060680/s1.
