# Supplementary material for: Two New Alkaloids and a Triterpenoid Glycoside from Rosa roxburghii with Antioxidant and Enzyme Inhibitory Activities
Source: Antioxidants (Basel). 2026 May 28;15(6):680. doi: 10.3390/antiox15060680 (PMC13296243; doi:10.3390/antiox15060680)

Supporting Information

Two New Alkaloids and a Triterpenoid Glycoside from Rosa roxburghii with Antioxidant and Enzyme Inhibitory Activities

Lang Zhou [a, b†], Yin-ju Zhang [a, b, c†], Wen-xia Dai [c], Fa-ju Chen [a, b], Xiong Pan [a, b], Yu Wang [a, b], Li-shou Yang [a, b], Qi-ji Li [a, b*], Xiao-Sheng Yang [a, b*]

[a] *State Key Laboratory of Discovery and Utilization of Functional Components in Traditional Chinese Medicine, Guizhou Medical University, Guiyang 550014, China.*

[b] *Natural Products Research Center of Guizhou Province, Guiyang, 550014, China.*

[C] *Guizhou University of Traditional Chinese Medicine, Guiyang, 550025, China*

[†]These authors contributed equally.

*Corresponding author.

E-mail address: leeqiji@126.com (Q. J. Li). gzcnp@sina.cn (X. S. Yang).

Table of Contents

Figure S1. ^{1}H-NMR (600 MHz, DMSO) spectrum of roxburghcid D (1). 1

Figure S2. ^{13}C-NMR (150 MHz, DMSO) spectrum of roxburghcid D (1). 1

Figure S3. HSQC spectrum of roxburghcid D (1)... 2

Figure S4. HMBC spectrum of roxburghcid D (1)... 2

Figure S5. ^{1}H-^{1}H COSY spectrum of roxburghcid D (1). 3

Figure S6. NOESY spectrum of roxburghcid D (1). 3

Figure S7. HR-ESI-MS spectrum of roxburghcid D (1). 4

Figure S8. UV spectrum of roxburghcid D (1)... 4

Figure S9. IR spectrum of roxburghcid D (1). 5

Figure S10. ^{1}H-NMR (600 MHz, DMSO) spectrum of roxburghcid E (2)......................... 5

Figure S11. ^{13}C-NMR (150 MHz, DMSO) spectrum of roxburghcid E (2)......................... 6

Figure S12. HSQC spectrum of roxburghcid E (2). 6

Figure S13. HMBC spectrum of roxburghcid E (2). 7

Figure S14. ^{1}H-^{1}H COSY spectrum of roxburghcid E (2)... 7

Figure S15. NOESY spectrum of roxburghcid E (2).. 8

Figure S16. HR-ESI-MS spectrum of roxburghcid E (2).. 8

Figure S17. UV spectrum of roxburghcid E (2). 9

Figure S18. IR spectrum of roxburghcid E (2). 9

Figure S19. ^{1}H-NMR (600 MHz, CD_3OD) spectrum of aegelbine-A (**3**). 10

Figure S20. ^{13}C-NMR (150 MHz, CD_3OD) spectrum of aegelbine-A (**3**). 10

Figure S21. ^{1}H-NMR (600 MHz, $CDCl_3$) spectrum of canthine-6-one (**4**)....................... 11

Figure S22. ^{13}C-NMR (150 MHz, $CDCl_3$) spectrum of canthine-6-one (**4**)...................... 11

Figure S23. ^{1}H-NMR (600 MHz, CD_3OD) spectrum of (*R*)-5-(1-hydroxyethyl)-canthine-6-one (**5**).. 12

Figure S24. ^{13}C-NMR (150 MHz, CD_3OD) spectrum of (*R*)-5-(1-hydroxyethyl)-canthine-6-one (**5**). 12

Figure S25. ^{1}H-NMR (800 MHz, DMSO) spectrum of roxburghcid C (**6**)....................... 13

Figure S26. ^{13}C-NMR (200 MHz, DMSO) spectrum of roxburghcid C (**6**)...................... 13

Figure S27. HSQC spectrum of roxburghcid C (6). 14

Figure S28. HMBC spectrum of roxburghcid C (6). 14

Figure S29. ^{1}H-^{1}H COSY spectrum of roxburghcid C (6). 15

Figure S30. NOESY spectrum of roxburghcid C (6). 15

Figure S31. HR-ESI-MS spectrum of roxburghcid C (6). 16

Figure S32. UV spectrum of roxburghcid C (6). 16

Figure S33. IR spectrum of roxburghcid C (6). 17

Figure S34. ^{1}H-NMR (600 MHz, CD_3OD) spectrum of 2*α*,3*β*,19*α*,23-tetrahydroxylurs-12-en-28-oic acid-*β*-D-glucopyranosyl ester (**7**). 17

Figure S35. ^{13}C-NMR (150 MHz, CD_3OD) spectrum of 2*α*,3*β*,19*α*,23-tetrahydroxylurs-12-en-28-oic acid-*β*-D-glucopyranosyl ester (**7**). 18

Figure S36. ^{1}H-NMR (600 MHz, CD_3OD) spectrum of 2*α*,3*α*,19*α*-trihydroxy-olean-12-en-28-oic acid-*β*-*D*-glucopyranosyl ester (**8**). 18

Figure S37. ^{13}C-NMR (150 MHz, CD_3OD) spectrum of 2*α*,3*α*,19*α*-trihydroxy-olean-12-en-28-oic acid-*β*-*D*-glucopyranosyl ester (**8**). 19

Figure S1. ^{1}H-NMR (600 MHz, DMSO) spectrum of roxburghcid D (**1**).

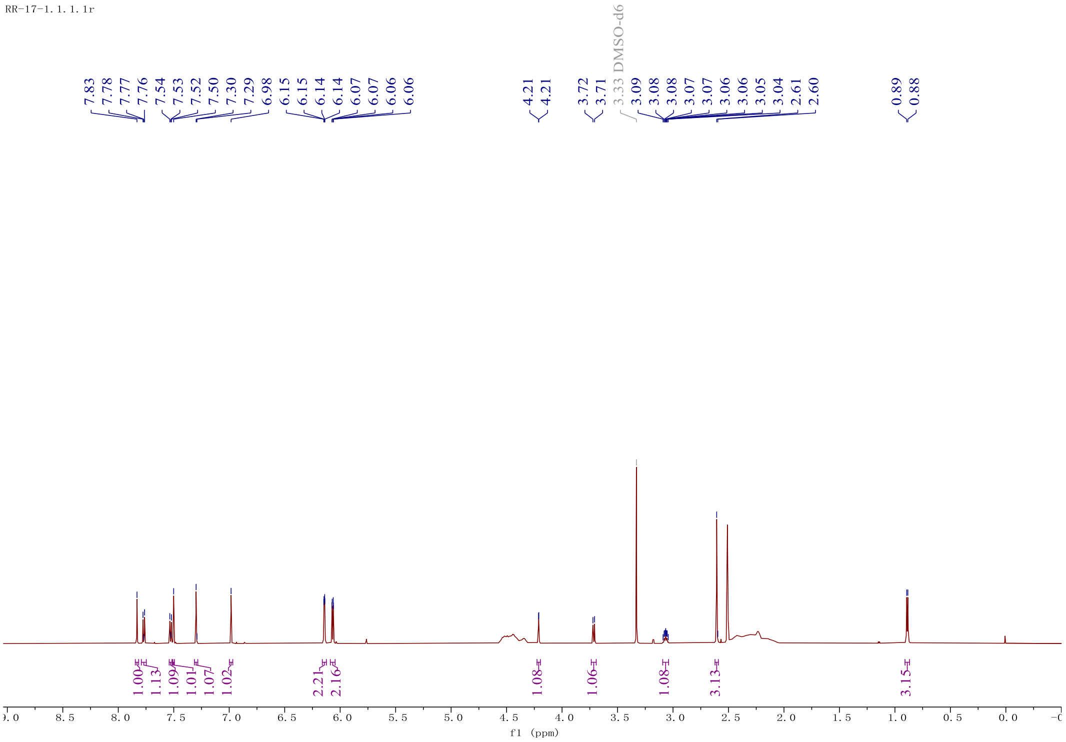

Figure S2. ^{13}C-NMR (150 MHz, DMSO) spectrum of roxburghcid D (**1**).

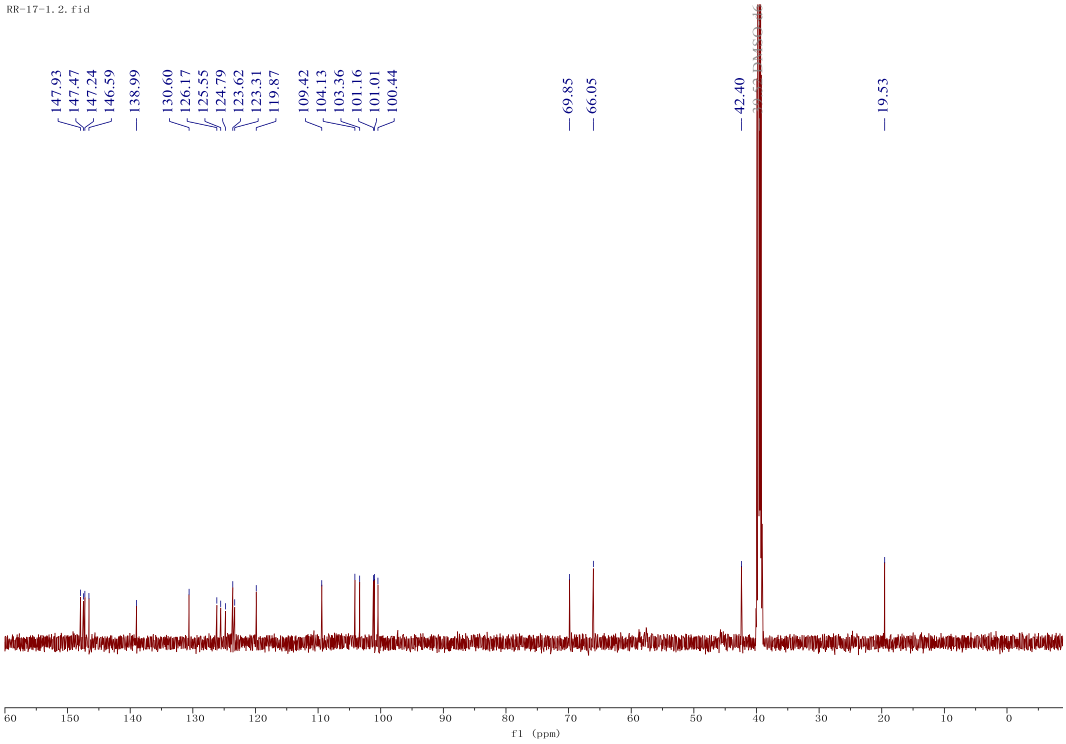

Figure S3. HSQC spectrum of roxburghcid D (**1**).

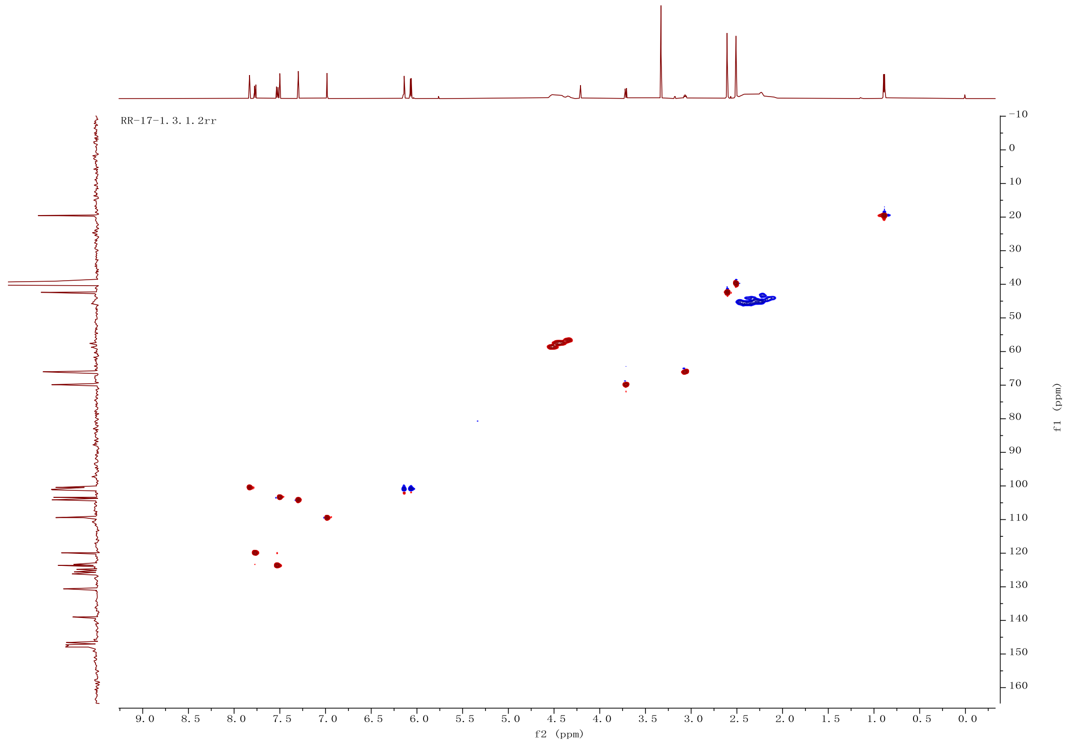

Figure S4. HMBC spectrum of roxburghcid D (**1**).

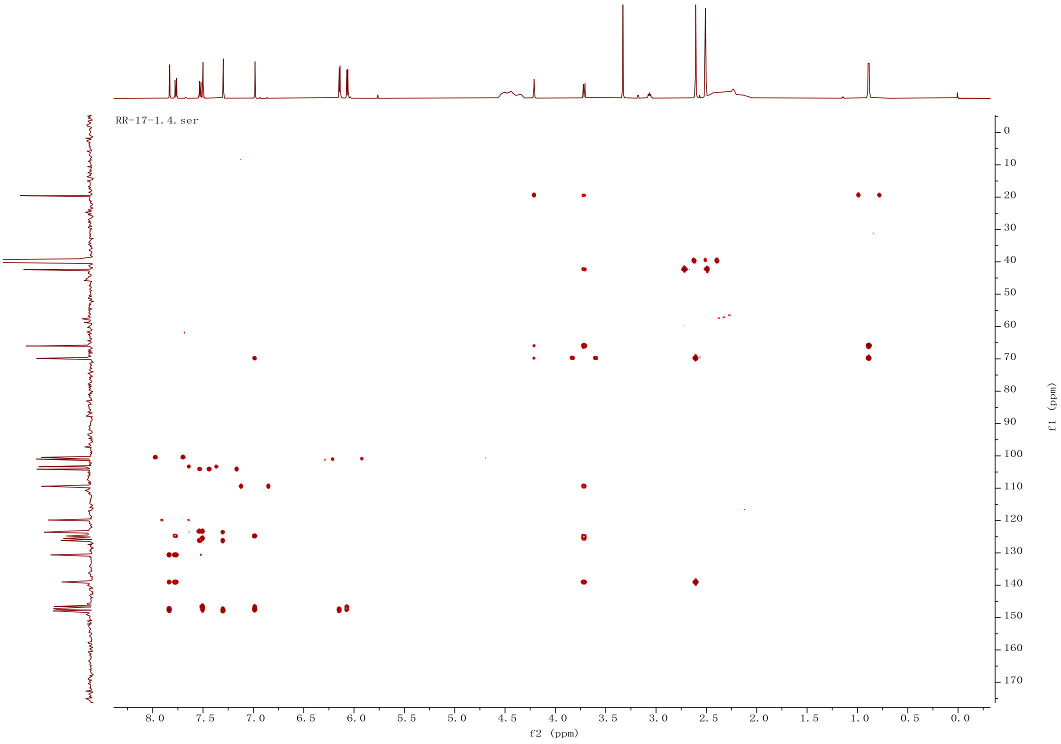

Figure S5. ^{1}H-^{1}H COSY spectrum of roxburghcid D (**1**).

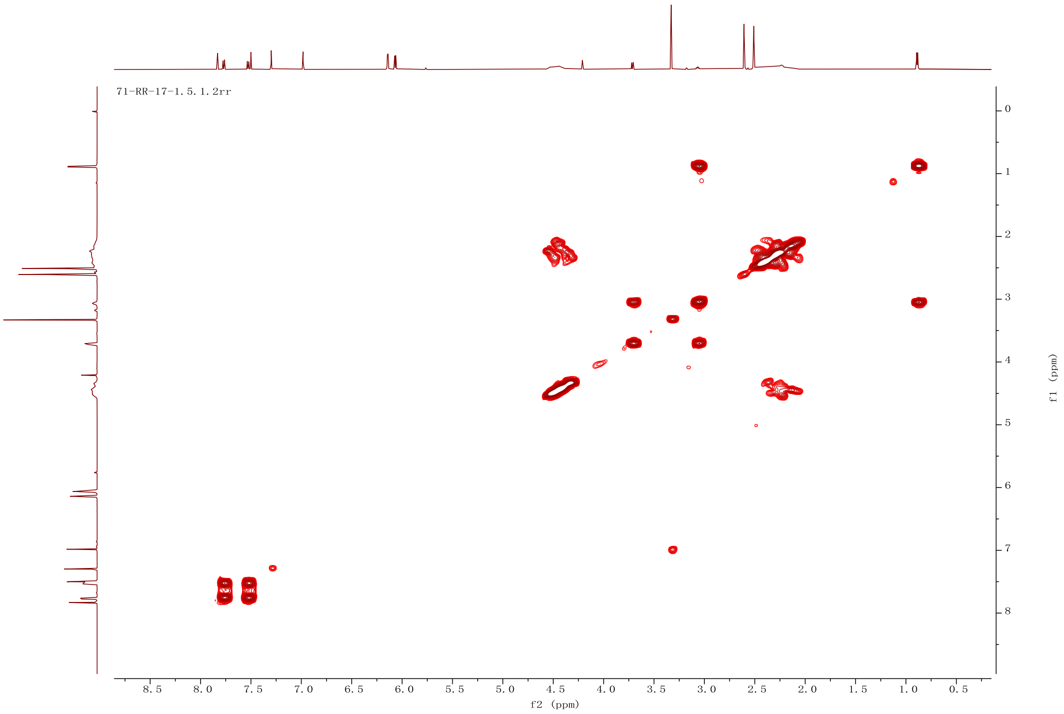

Figure S6. NOESY spectrum of roxburghcid D (**1**).

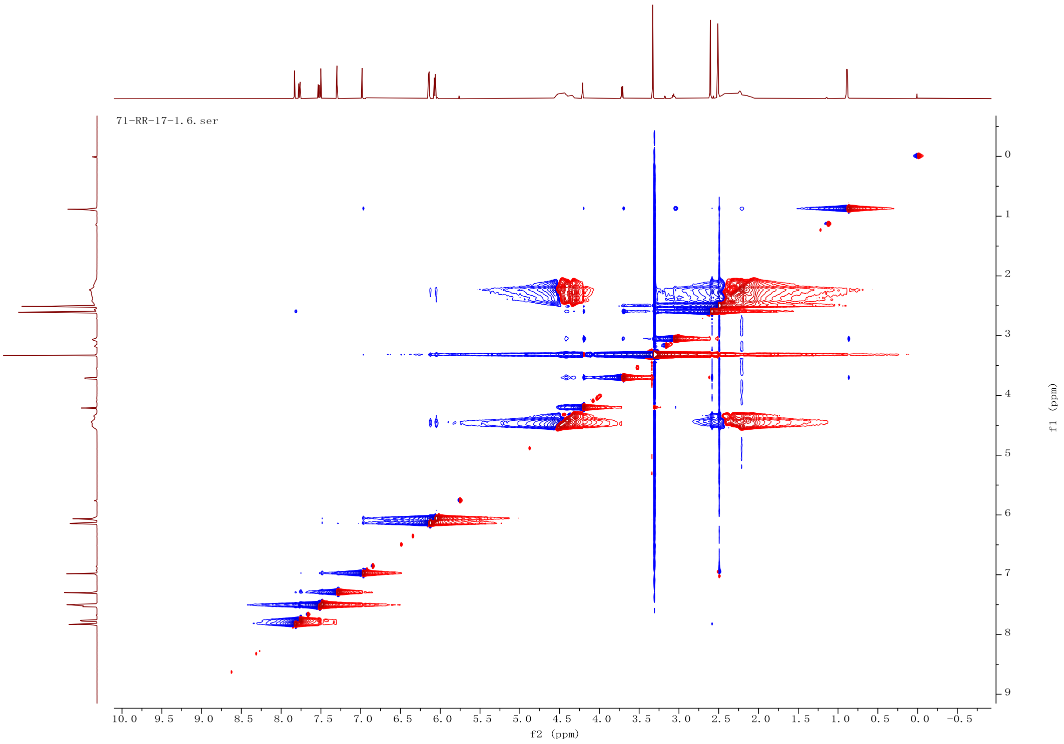

Figure S7. HR-ESI-MS spectrum of roxburghcid D (**1**).

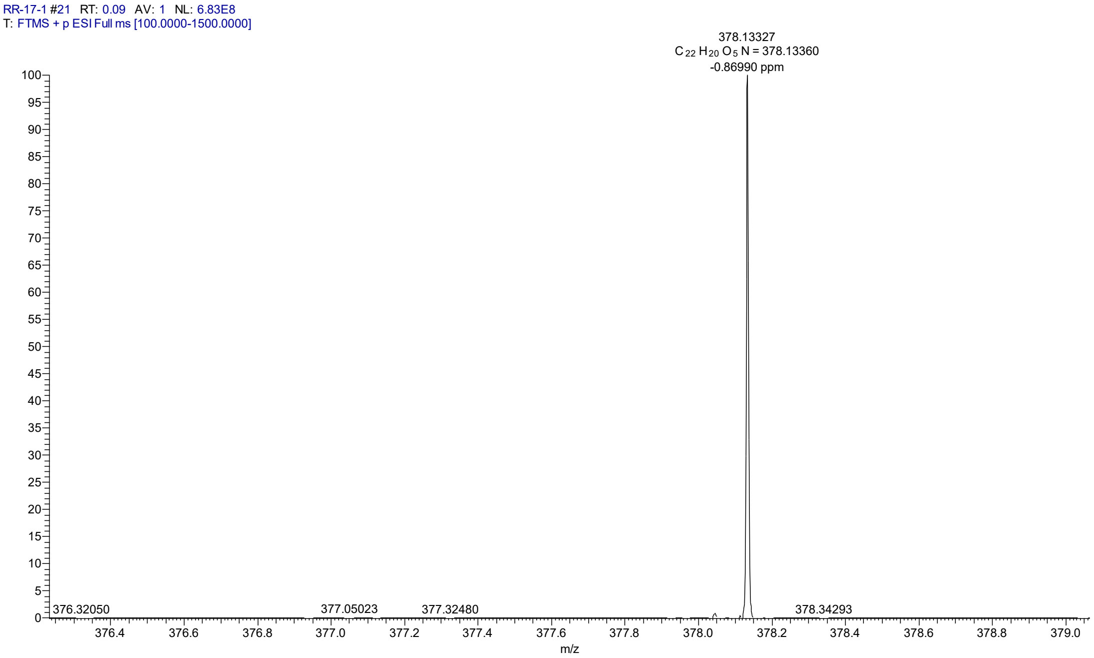

Figure S8. UV spectrum of roxburghcid D (**1**).

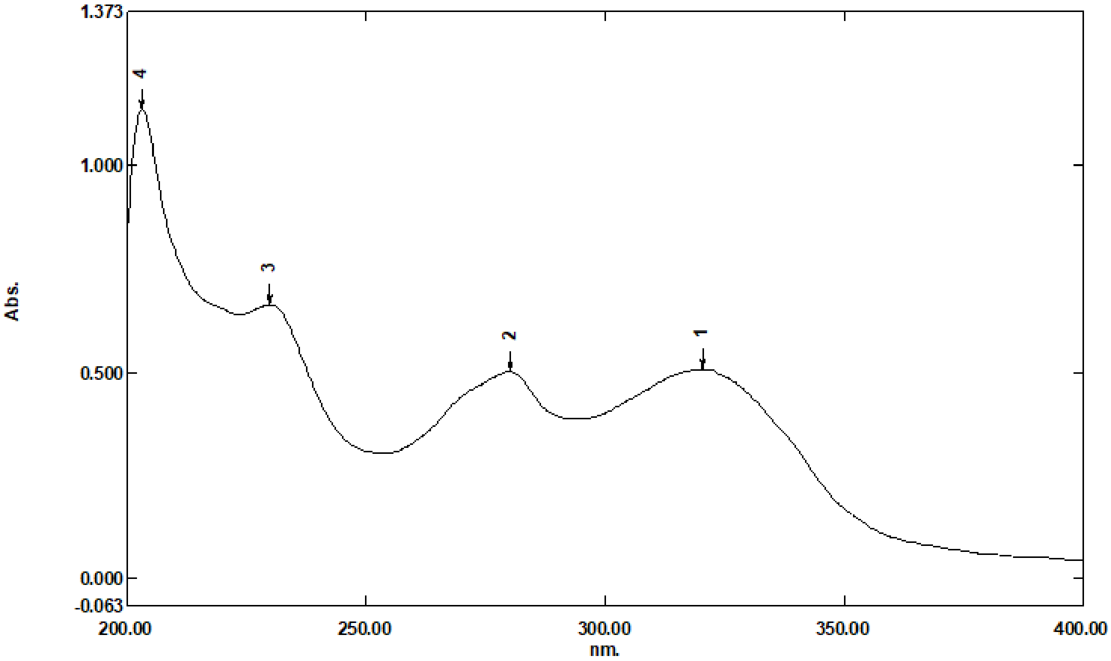

No. P/V	Wavelength(nm)	Abs.
1	320.40	0.506
2	280.00	0.501
3	229.80	0.664
4	203.00	1.136

Figure S9. IR spectrum of roxburghcid D (**1**).

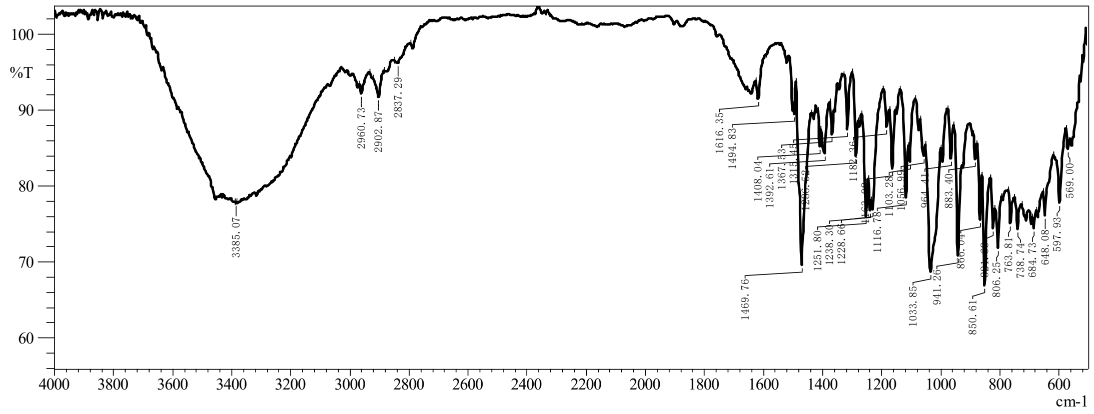

Figure S10. ^{1}H-NMR (600 MHz, DMSO) spectrum of roxburghcid E (**2**).

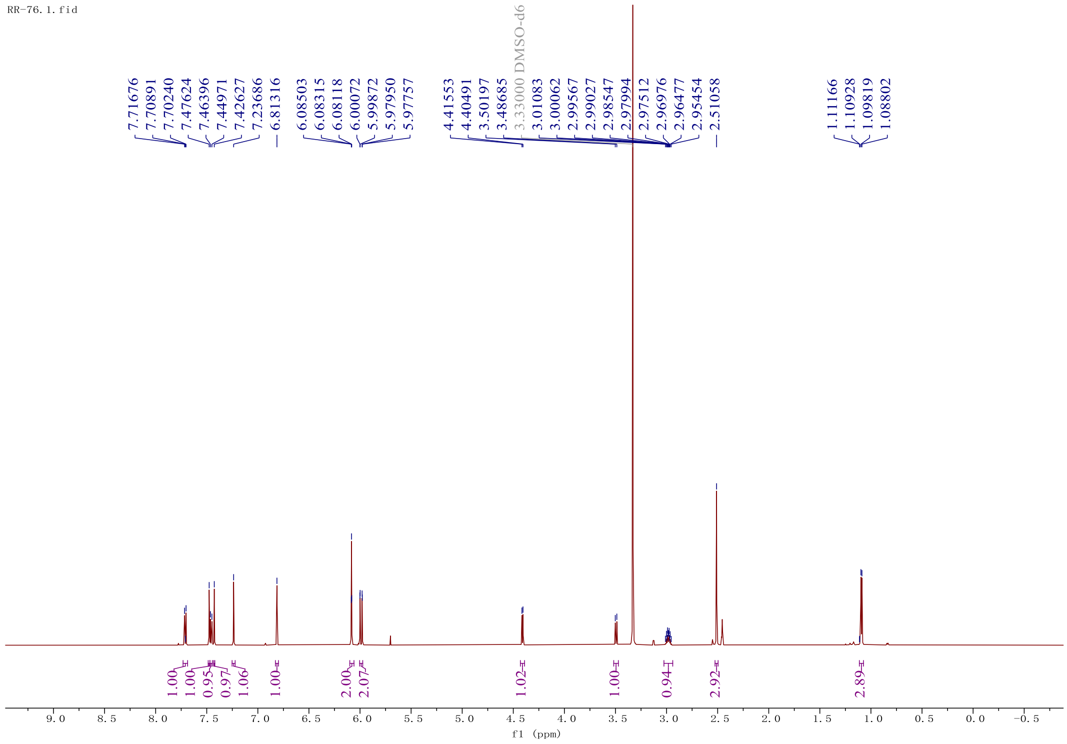

Figure S11. ^{13}C-NMR (150 MHz, DMSO) spectrum of roxburghcid E (**2**).

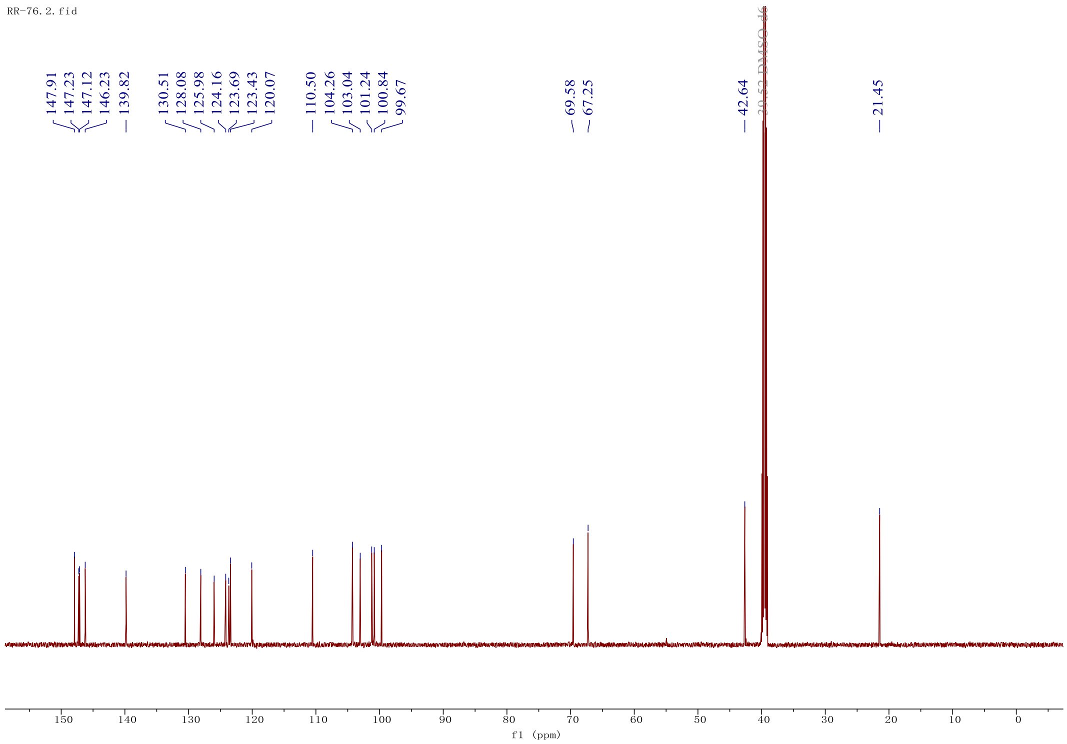

Figure S12. HSQC spectrum of roxburghcid E (**2**).

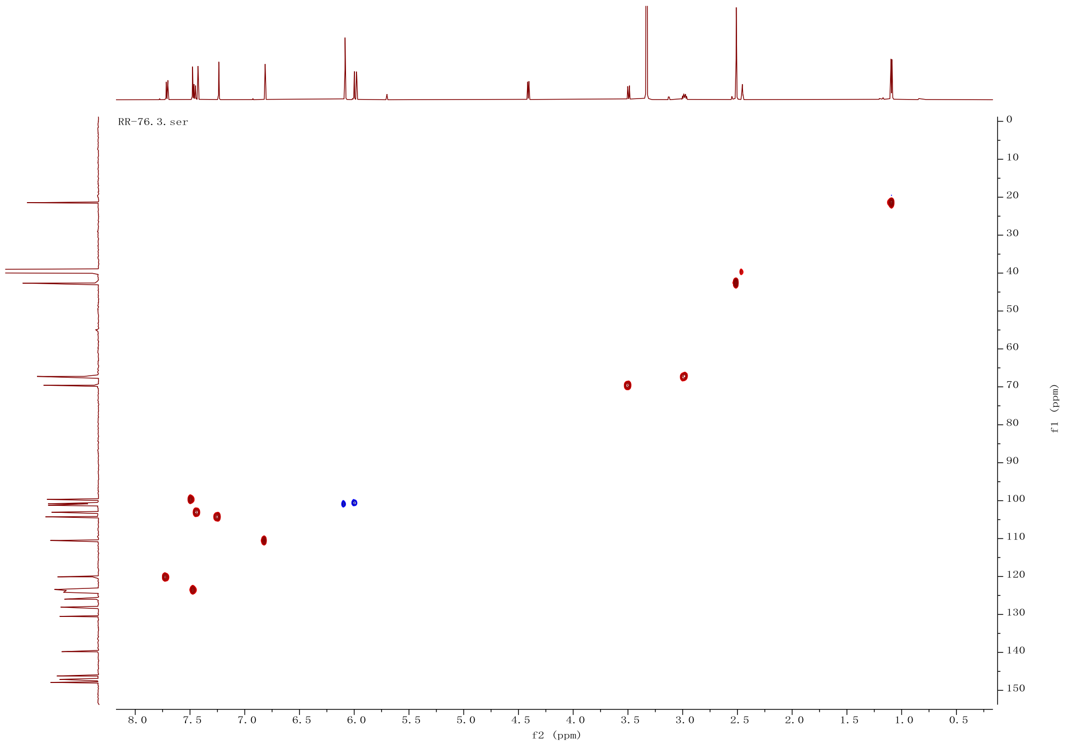

Figure S13. HMBC spectrum of roxburghcid E (**2**).

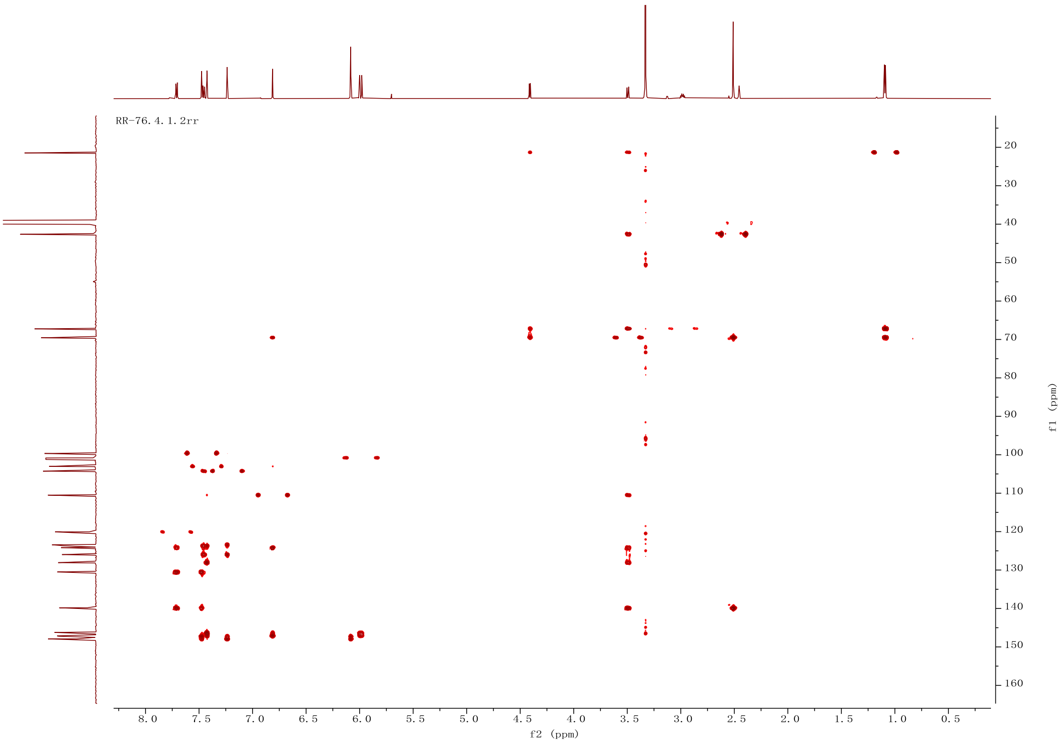

Figure S14. ^{1}H-^{1}H COSY spectrum of roxburghcid E (**2**).

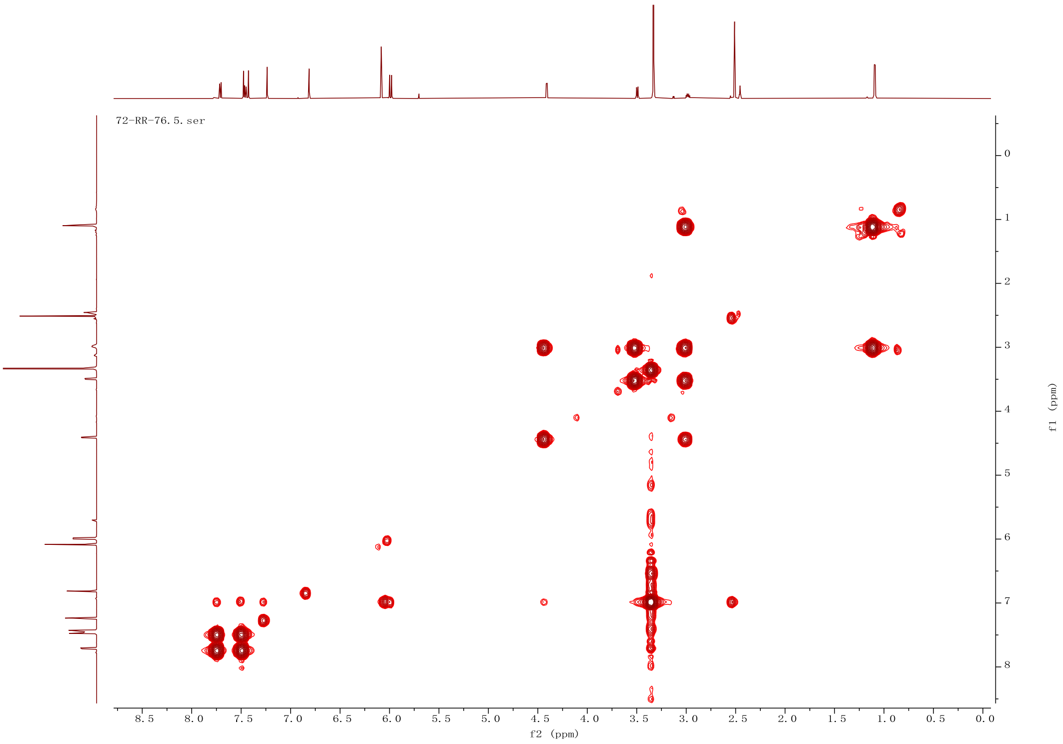

Figure S15. NOESY spectrum of roxburghcid E (**2**).

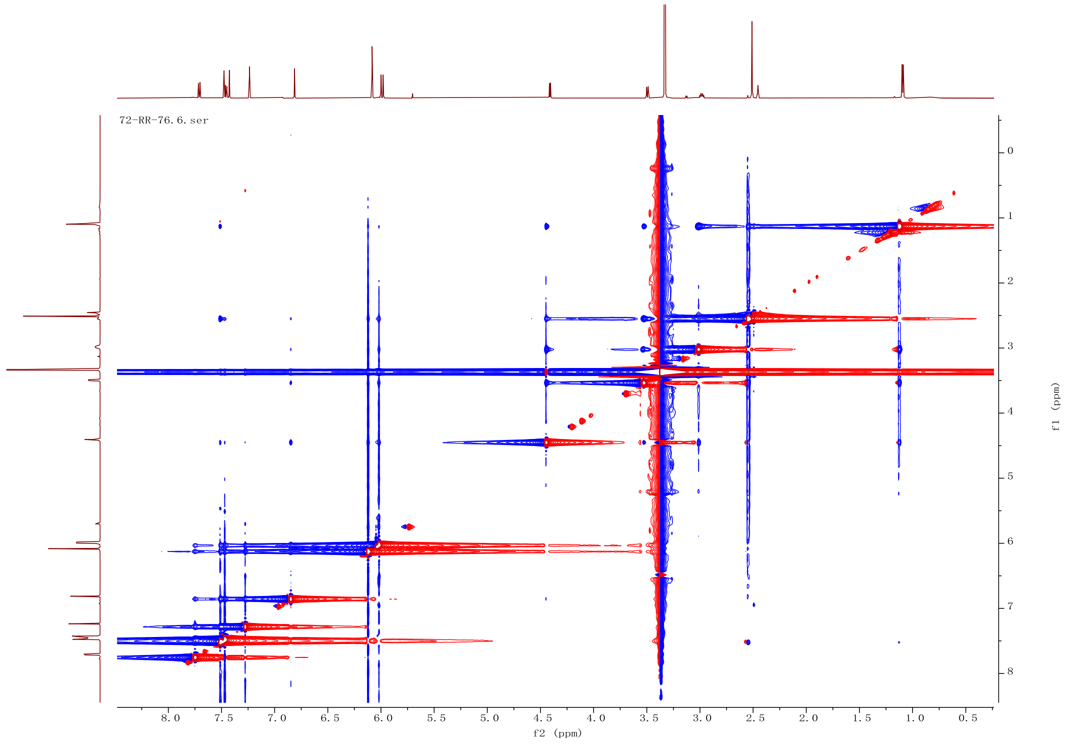

Figure S16. HR-ESI-MS spectrum of roxburghcid E (**2**).

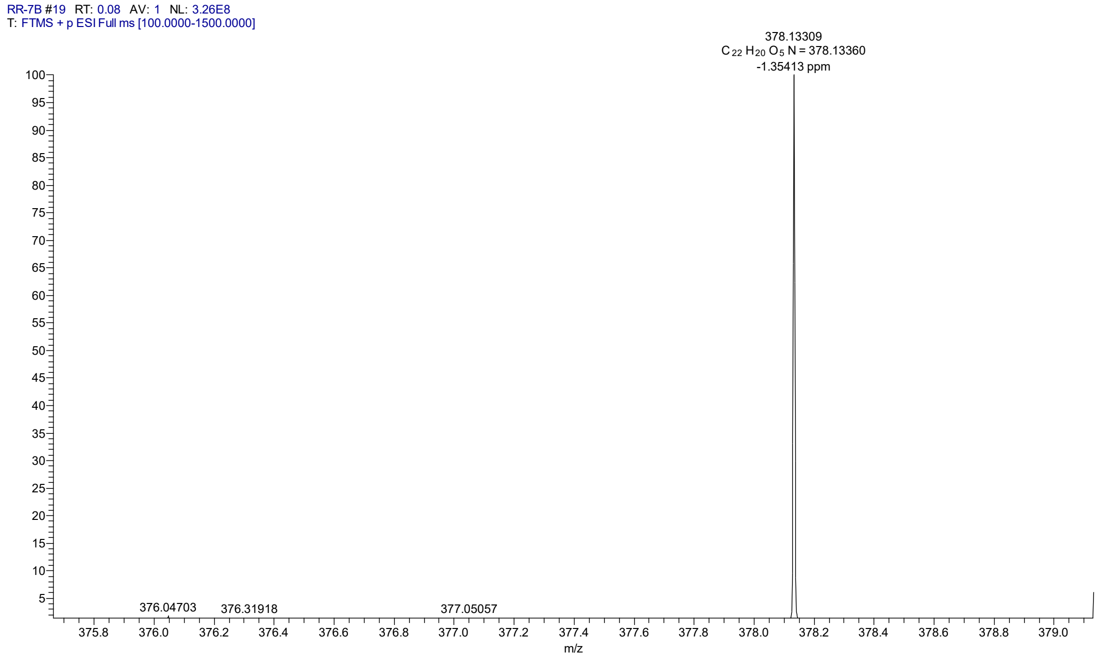

Figure S17. UV spectrum of roxburghcid E (**2**).

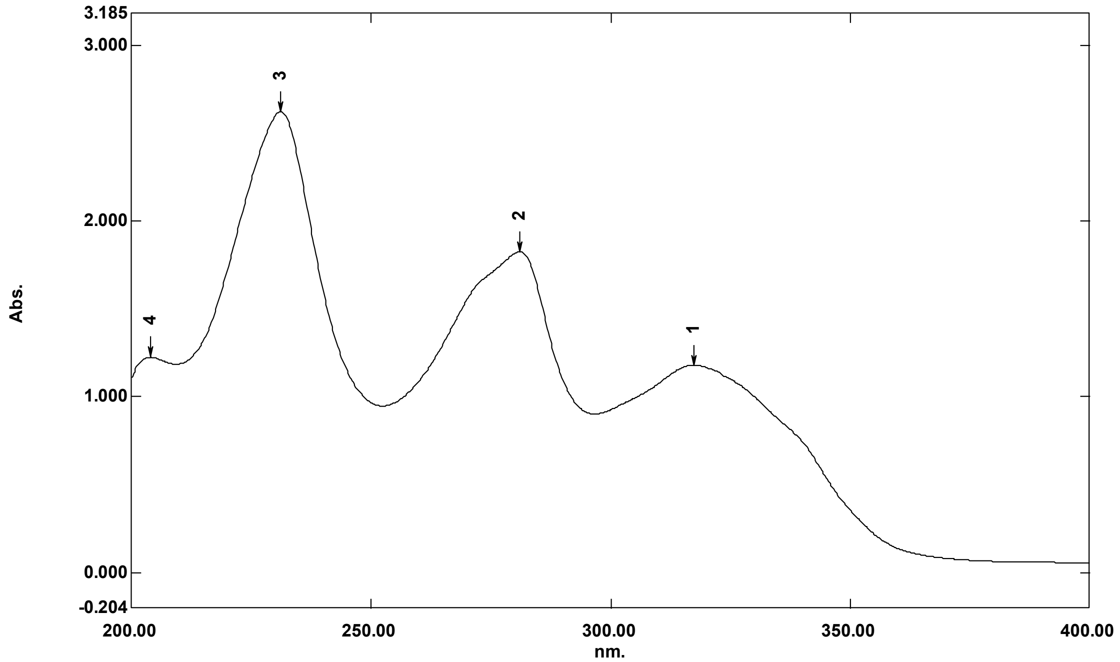

No.	P/V	Wavelength (nm)	Abs.
1		317.40	1.181
2		281.00	1.826
3		231.00	2.626
4		204.00	1.226

Figure S18. IR spectrum of roxburghcid E (**2**).

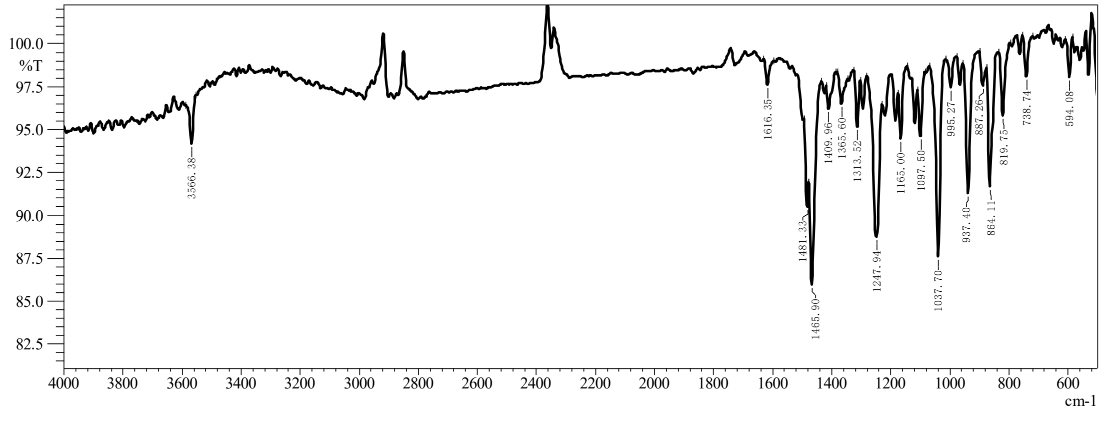

Figure S19. ^{1}H-NMR (600 MHz, CD_3OD) spectrum of aegelbine-A (**3**).

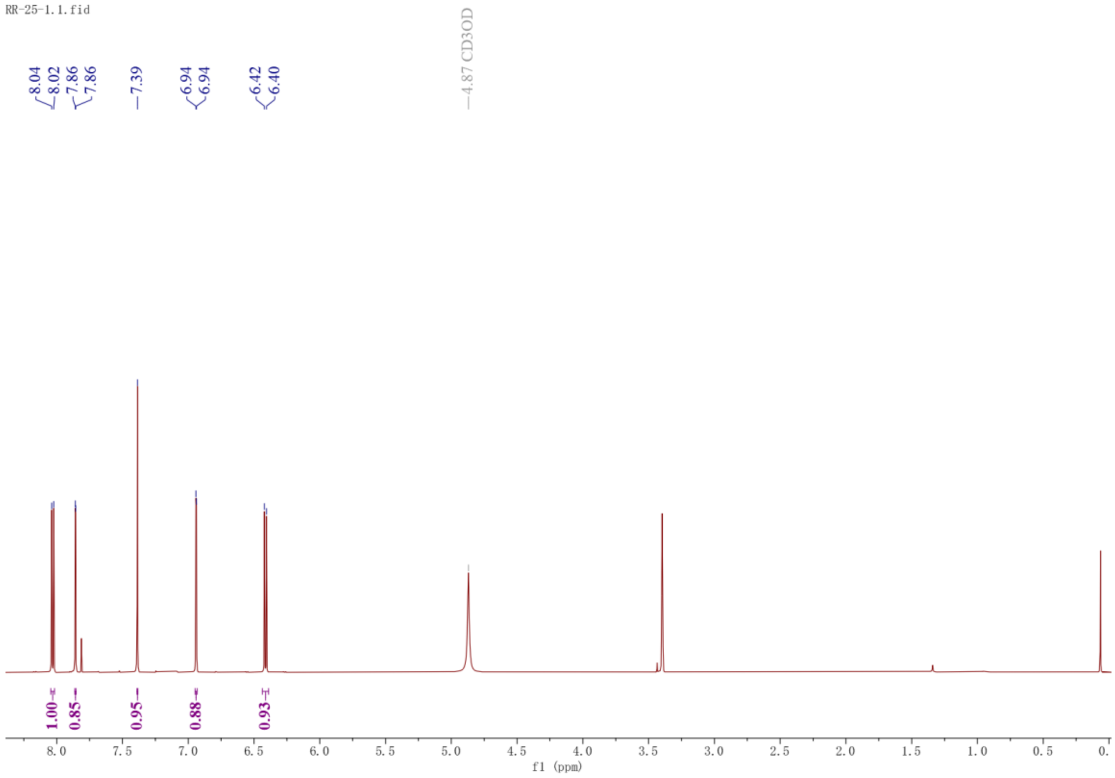

Figure S20. ^{13}C-NMR (150 MHz, CD_3OD) spectrum of aegelbine-A (**3**).

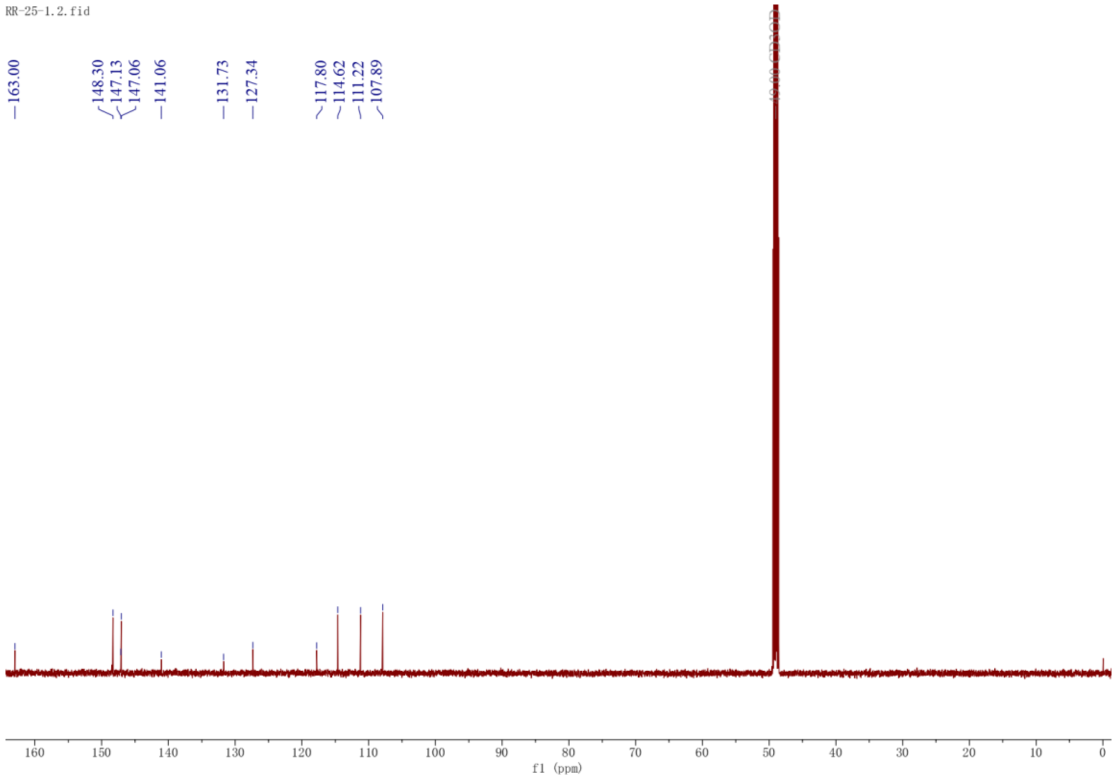

Figure S21. ^{1}H-NMR (600 MHz, $CDCl_3$) spectrum of canthine-6-one (**4**).

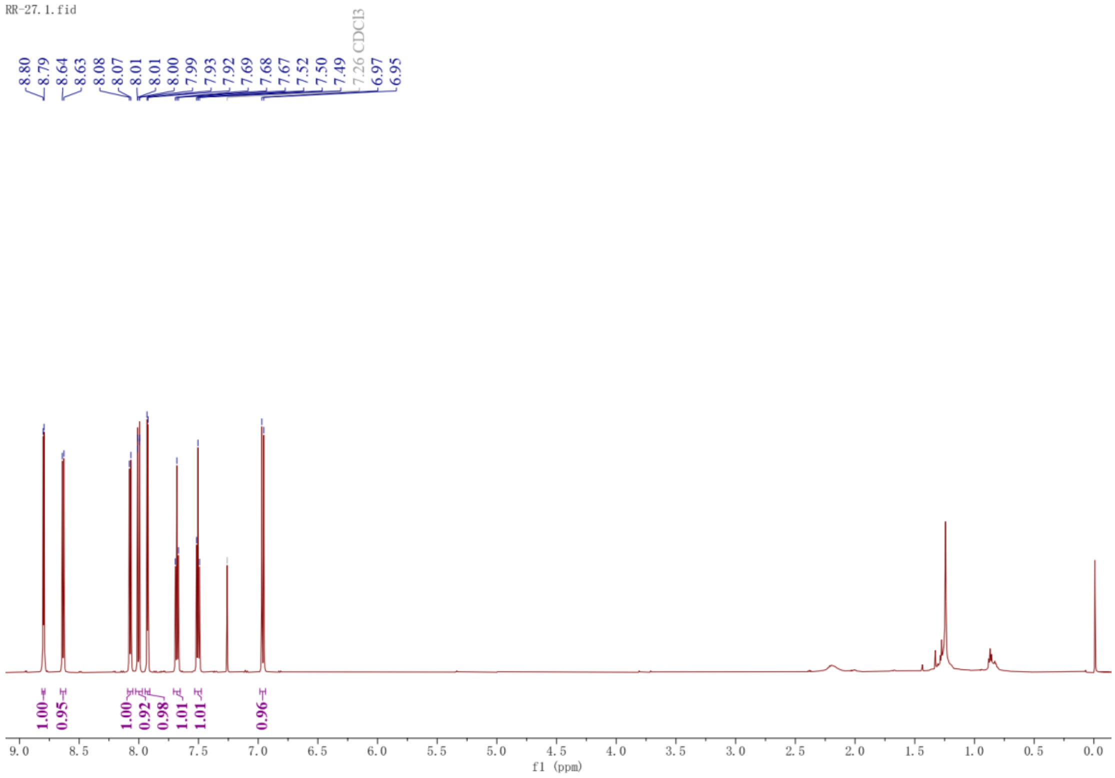

Figure S22. ^{13}C-NMR (150 MHz, $CDCl_3$) spectrum of canthine-6-one (**4**).

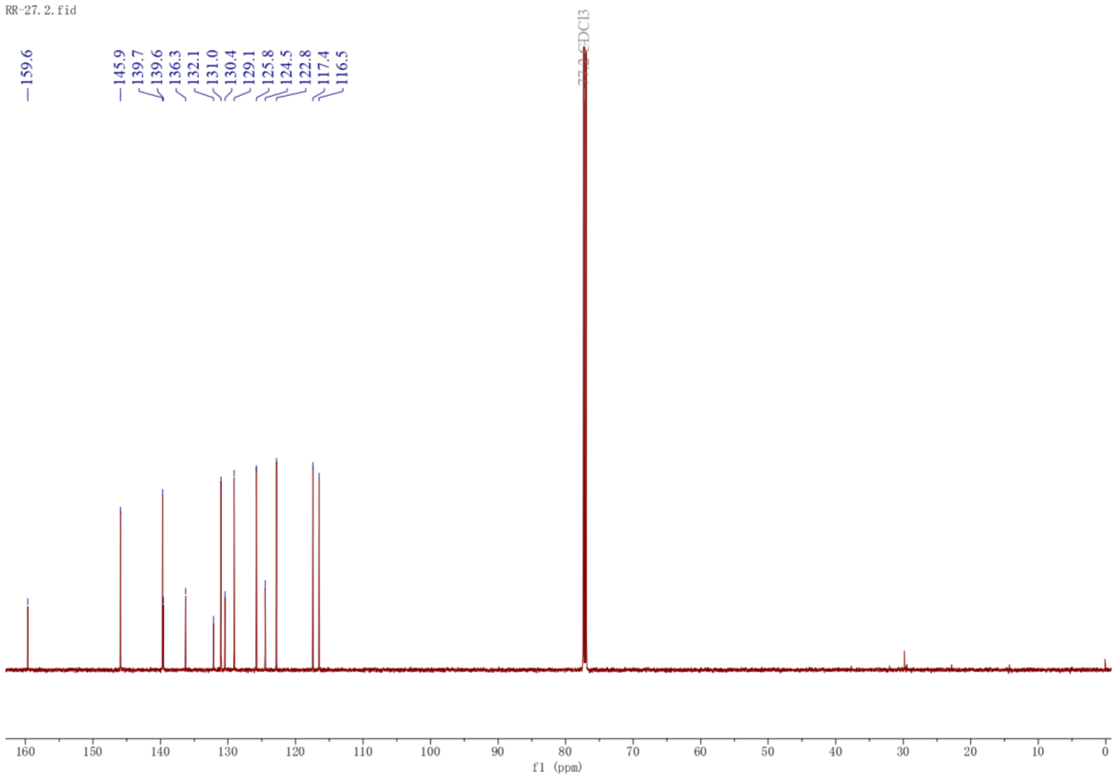

Figure S23. ^{1}H-NMR (600 MHz, CD_3OD) spectrum of (*R*)-5-(1-hydroxyethyl)-canthine-6-one (**5**).

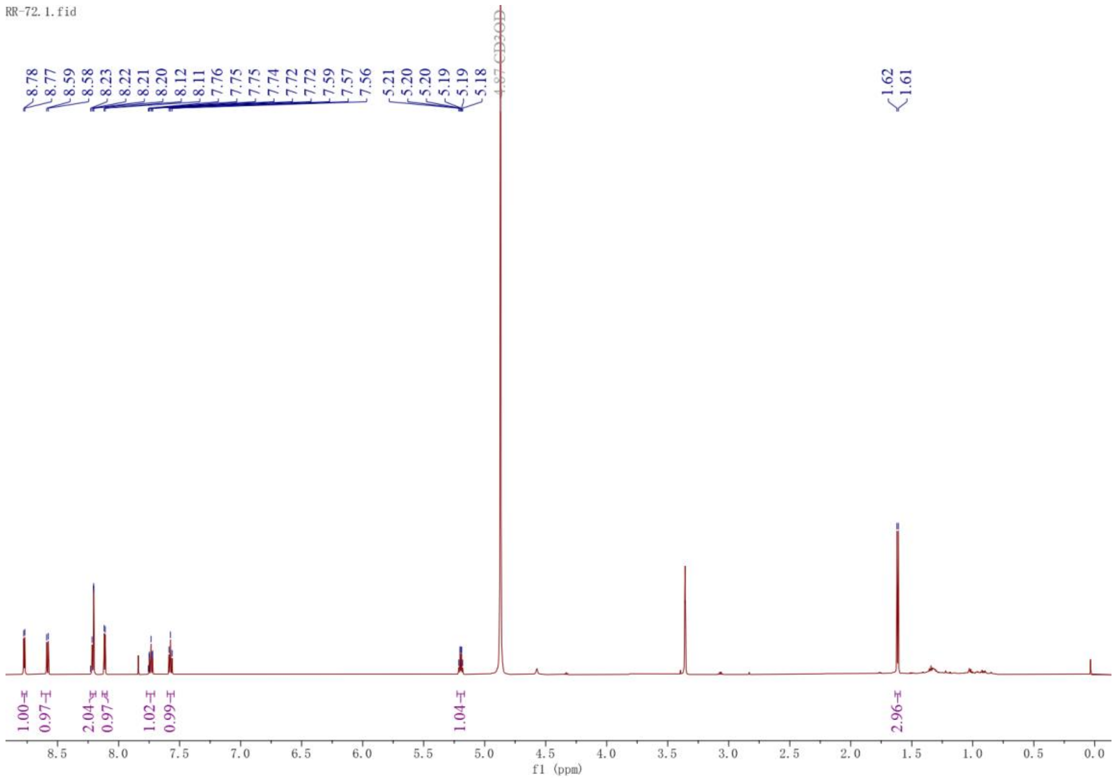

Figure S24. ^{13}C-NMR (150 MHz, CD_3OD) spectrum of (*R*)-5-(1-hydroxyethyl)-canthine-6-one (**5**).

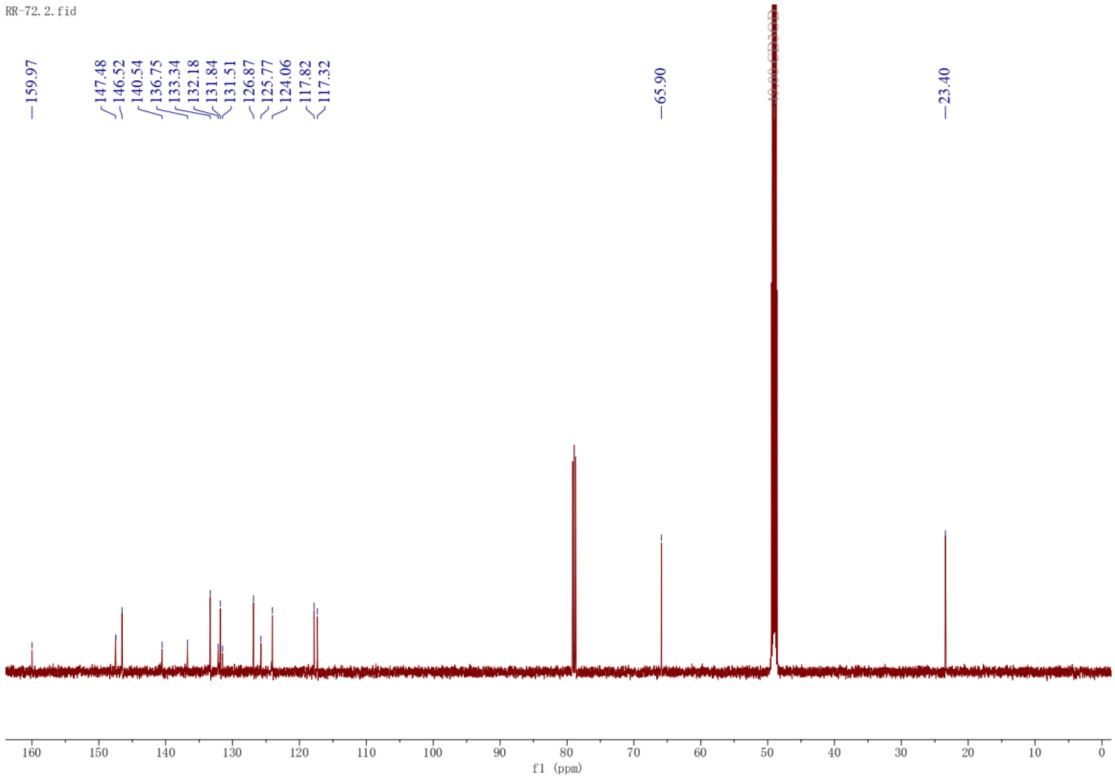

Figure S25. ^{1}H-NMR (800 MHz, DMSO) spectrum of roxburghcid C (**6**).

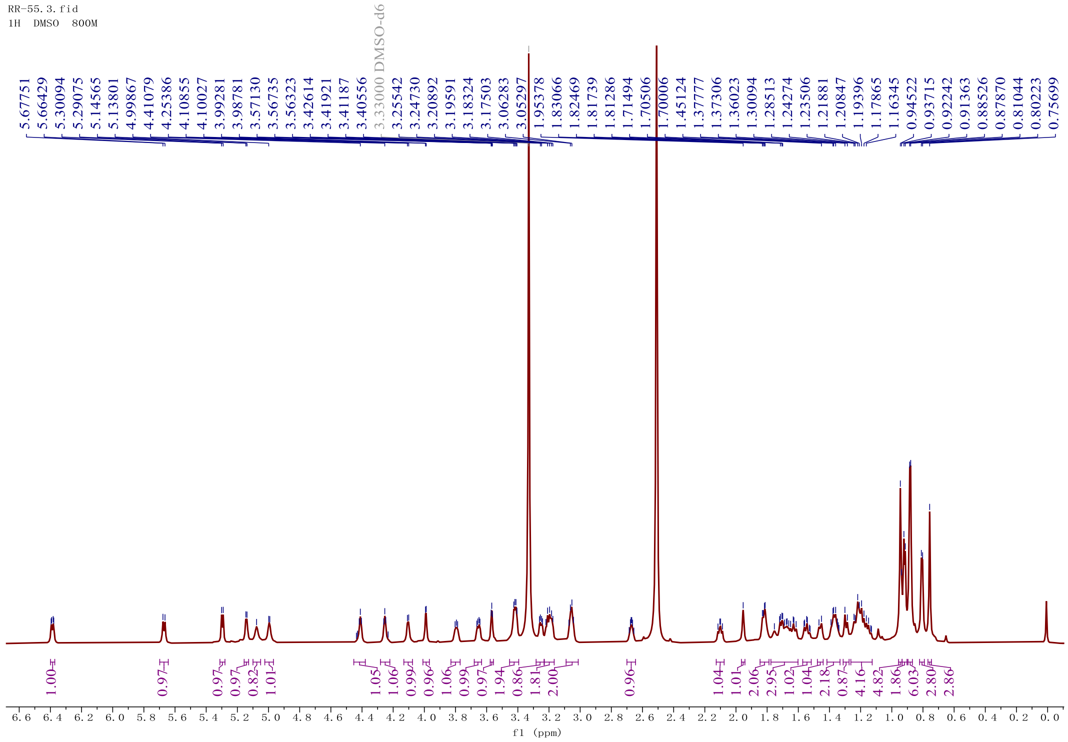

Figure S26. ^{13}C-NMR (200 MHz, DMSO) spectrum of roxburghcid C (**6**).

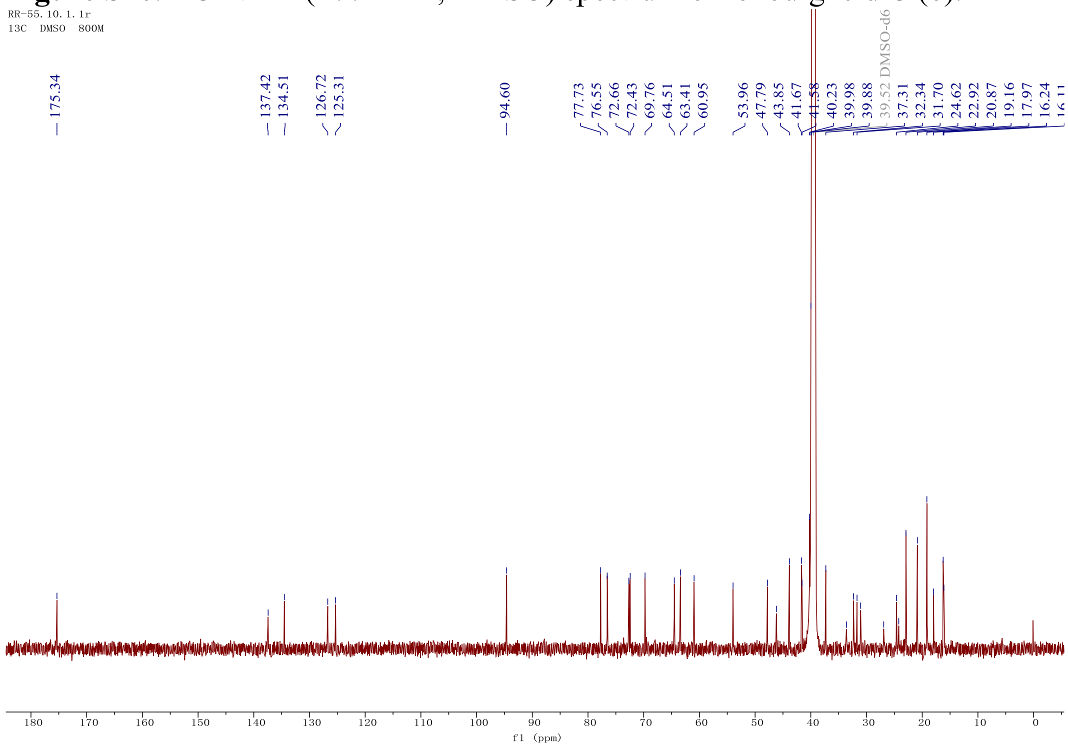

Figure S27. HSQC spectrum of roxburghcid C **(6)**.

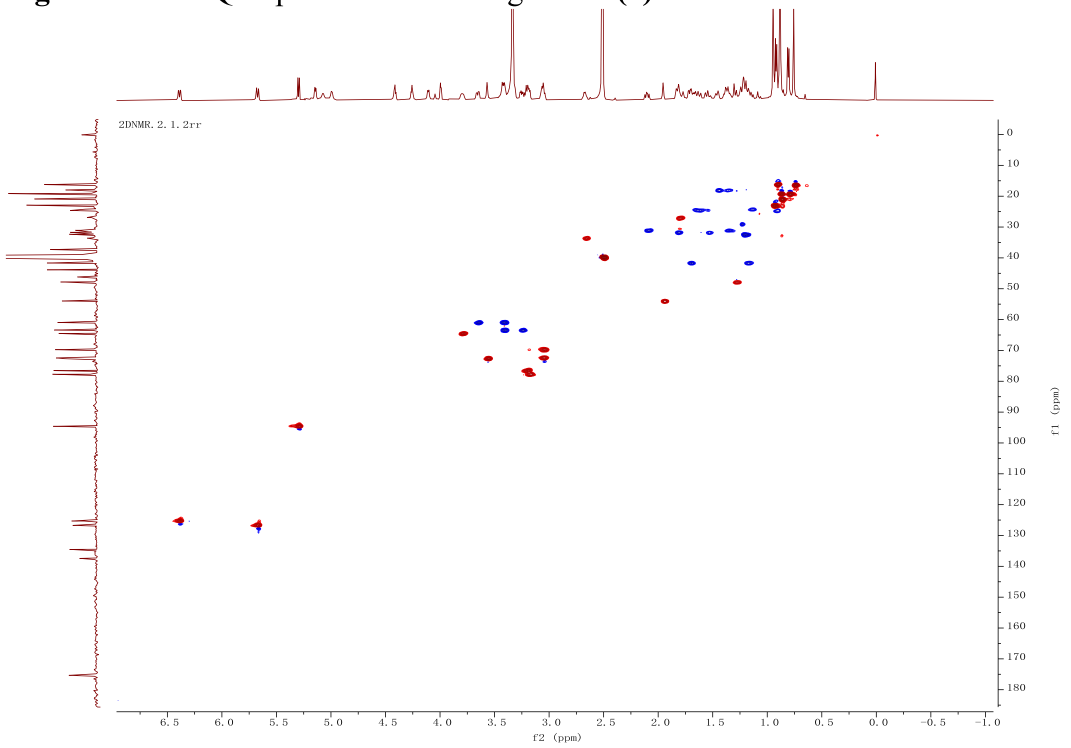

Figure S28. HMBC spectrum of roxburghcid C (**6**).

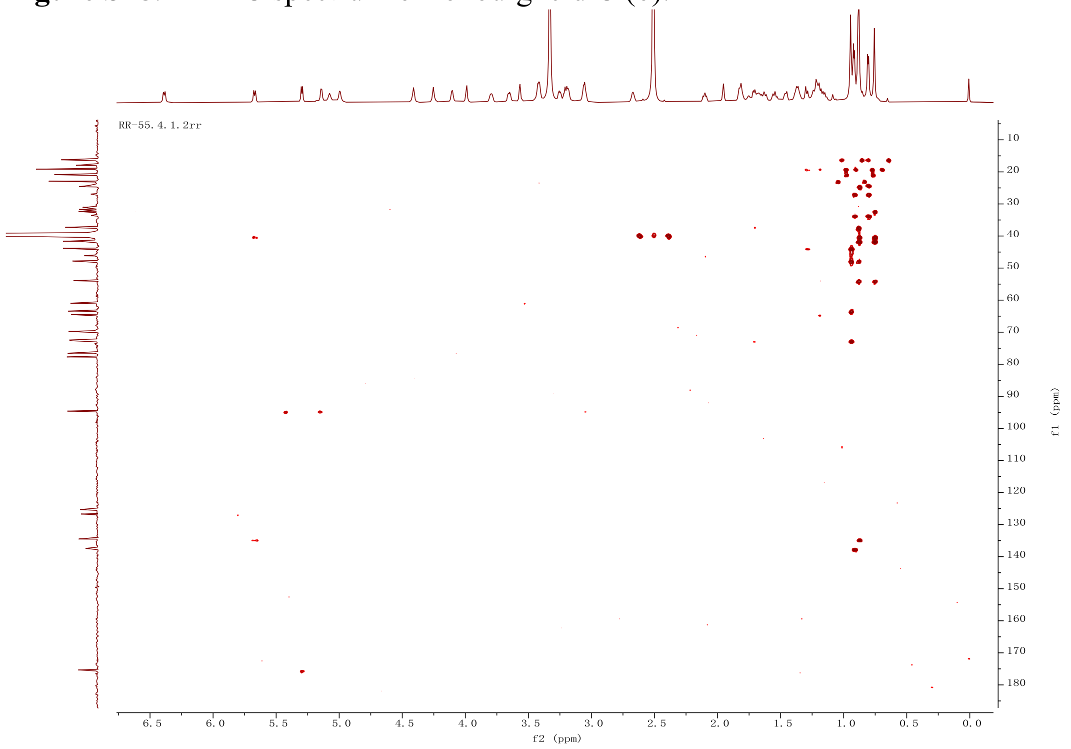

Figure S29. ^{1}H-^{1}H COSY spectrum of roxburghcid C (**6**).

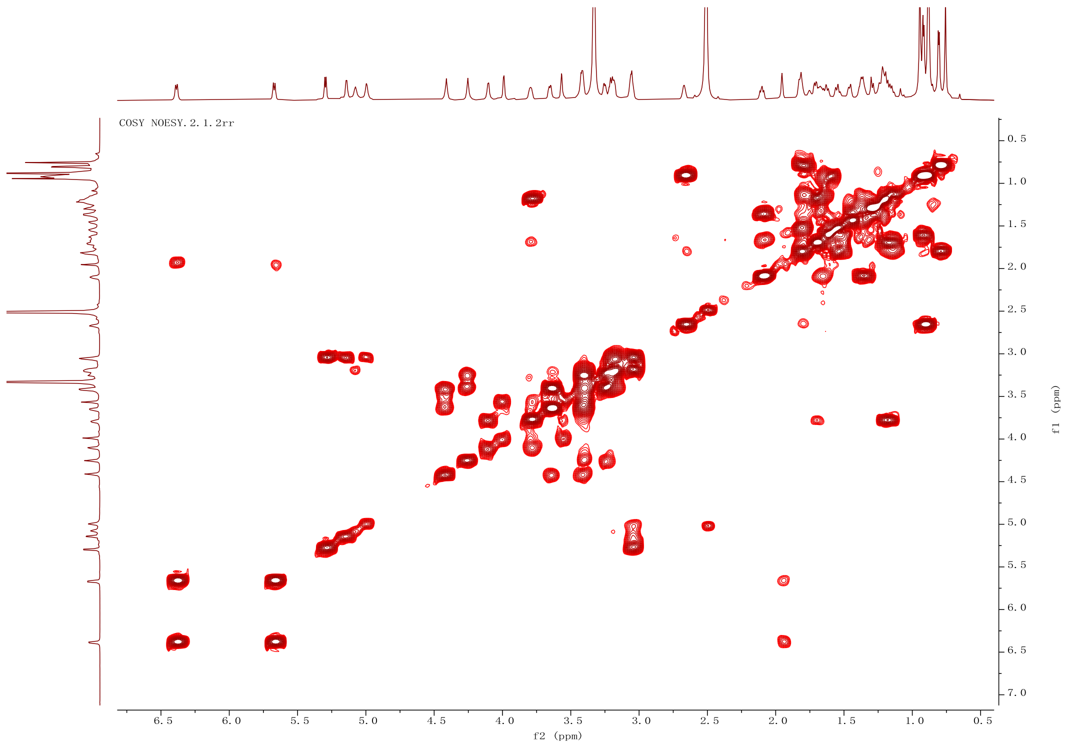

Figure S30. NOESY spectrum of roxburghcid C (**6**).

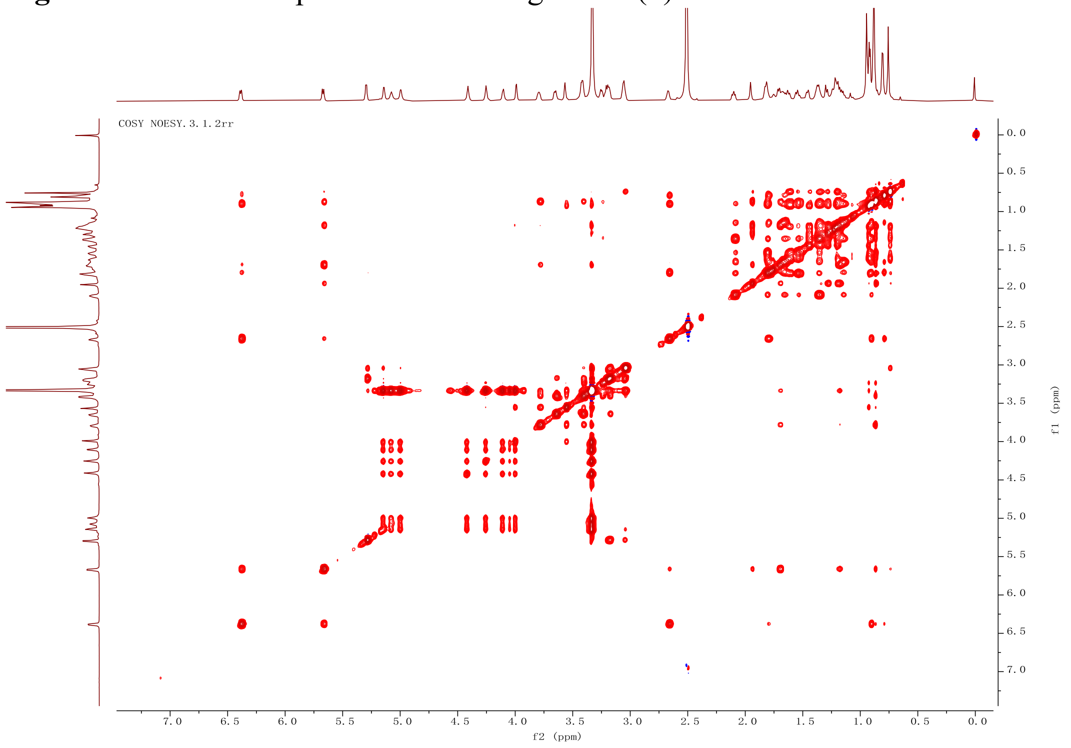

Figure S31. HR-ESI-MS spectrum of roxburghcid C (**6**).

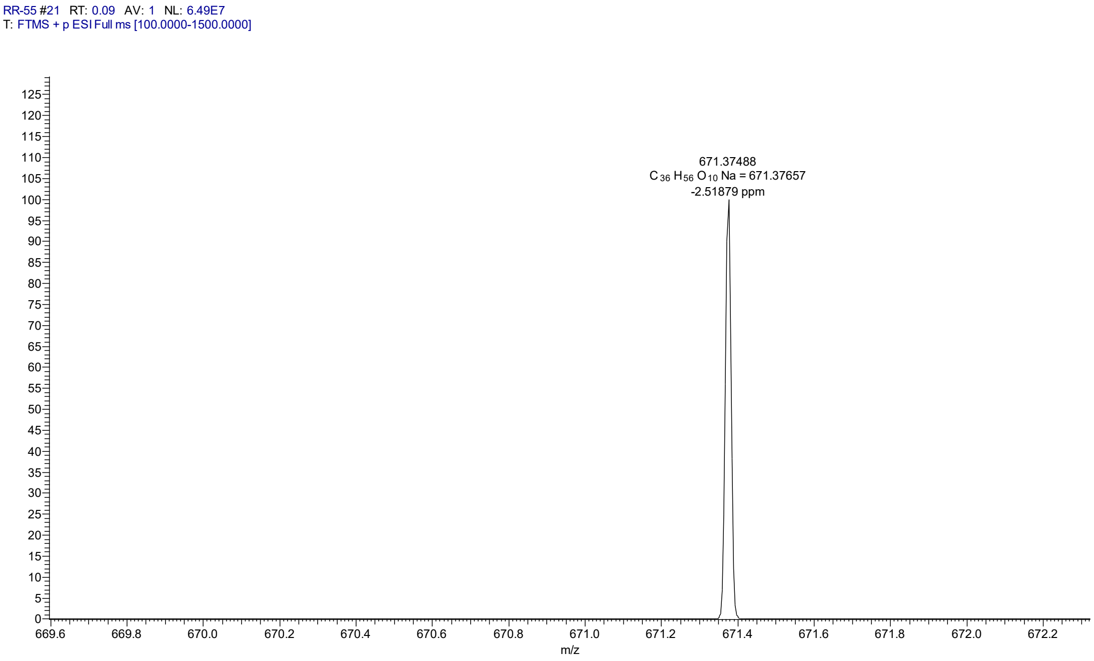

Figure S32. UV spectrum of roxburghcid C (**6**).

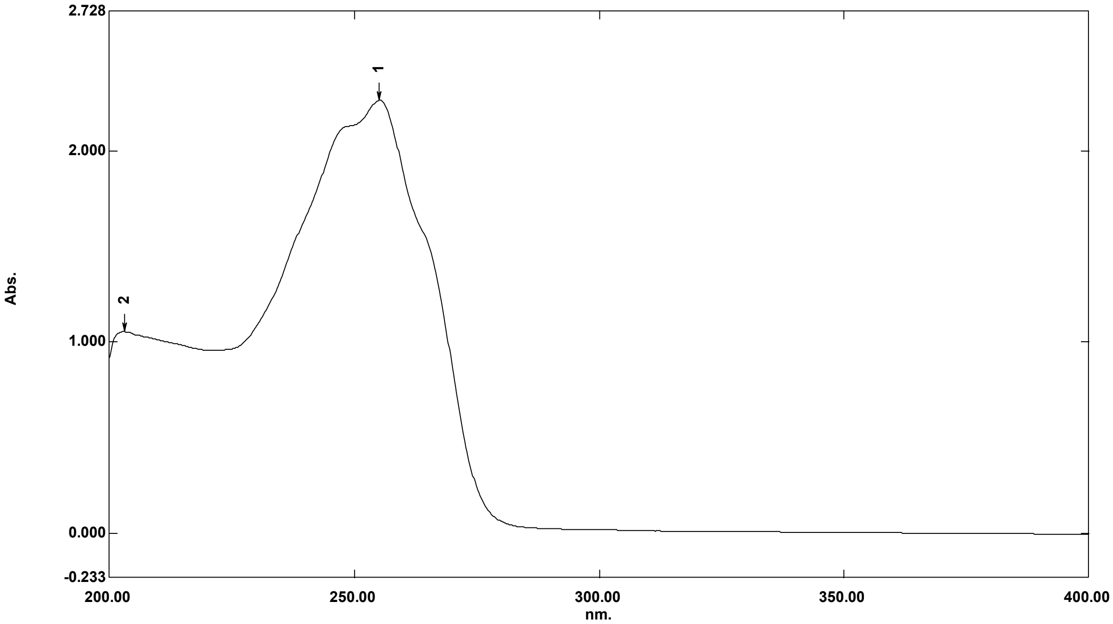

No.	P/V	Wavelength (nm)	Abs.
1		255.00	2.263
2		203.00	1.053

Figure S33. IR spectrum of roxburghcid C (**6**).

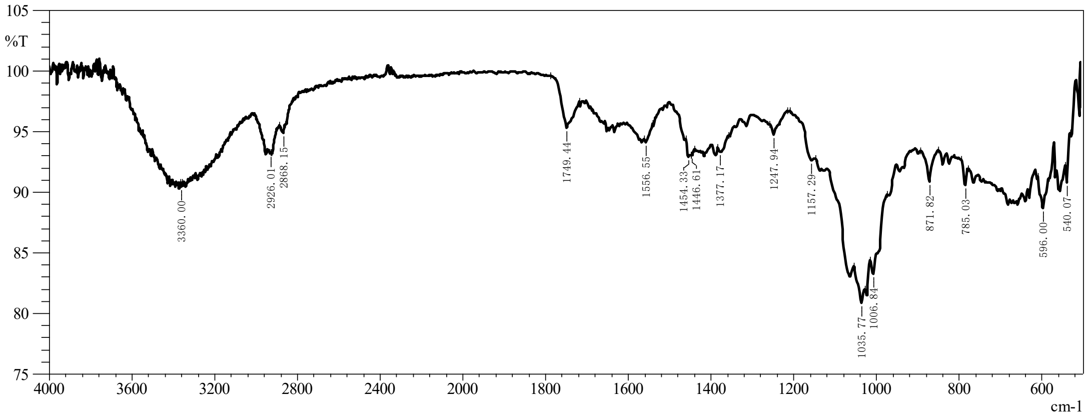

Figure S34. ^{1}H-NMR (600 MHz, CD_3OD) spectrum of 2*α*,3*β*,19*α*,23-tetrahydroxylurs-12-en-28-oic acid-*β*-D-glucopyranosyl ester (**7**).

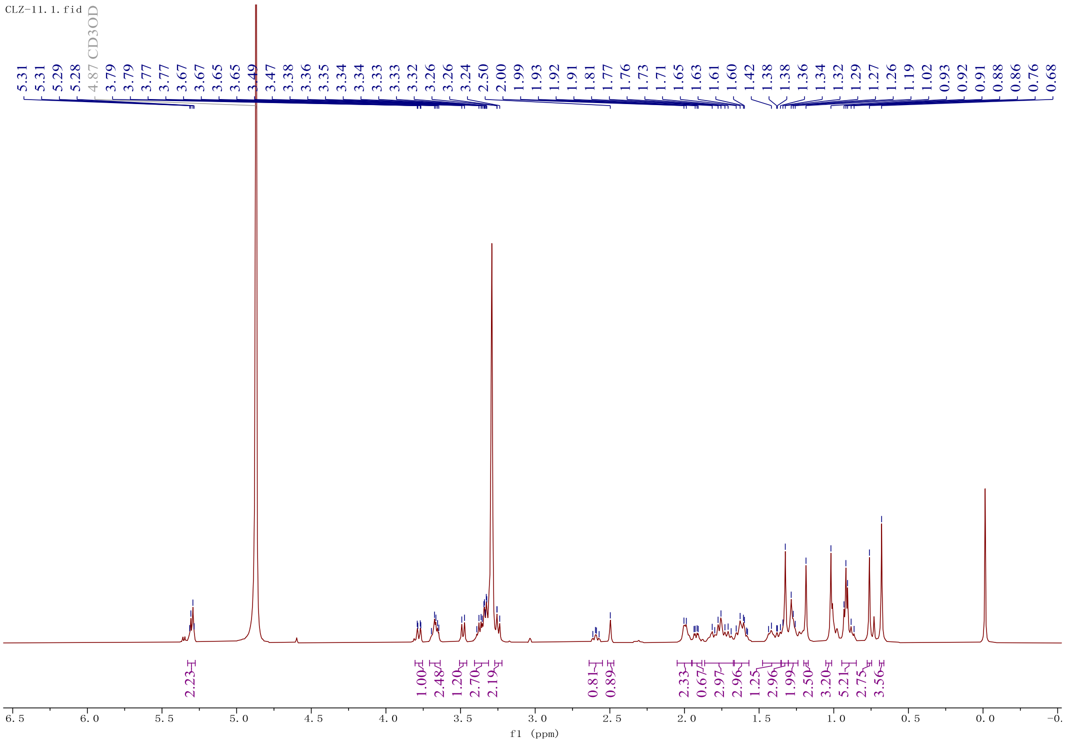

Figure S35. ^{13}C-NMR (150 MHz, CD_3OD) spectrum of 2*α*,3*β*,19*α*,23-tetrahydroxylurs-12-en-28-oic acid-*β*-D-glucopyranosyl ester (**7**).

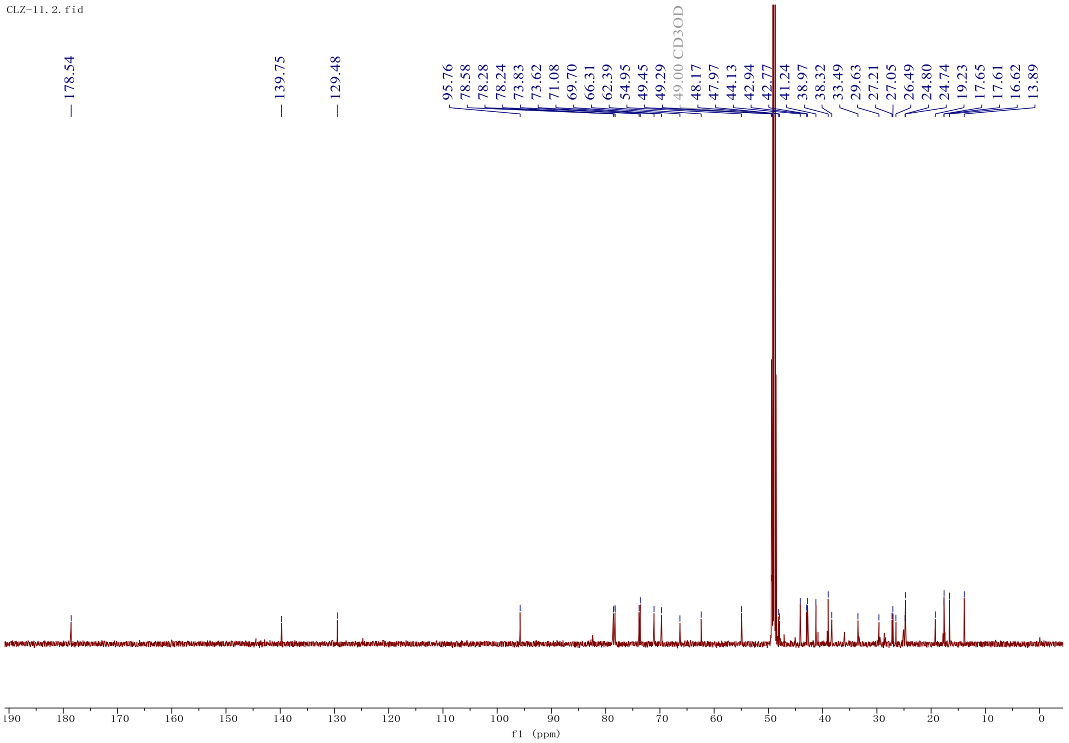

Figure S36. ^{1}H-NMR (600 MHz, CD_3OD) spectrum of 2*α*,3*α*,19*α*-trihydroxy-olean-12-en-28-oic acid-*β*-*D*-glucopyranosyl ester (**8**).

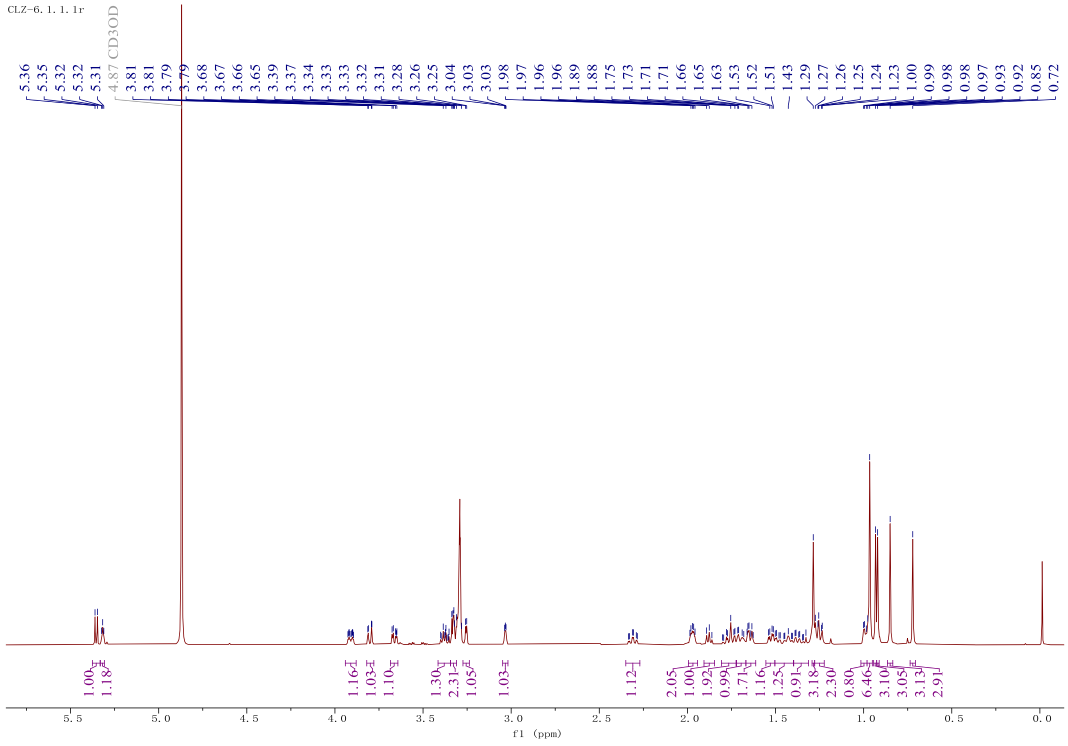

Figure S37. ^{13}C-NMR (150 MHz, CD_3OD) spectrum of 2*α*,3*α*,19*α*-trihydroxy-olean-12-en-28-oic acid-*β*-*D*-glucopyranosyl ester (**8**).

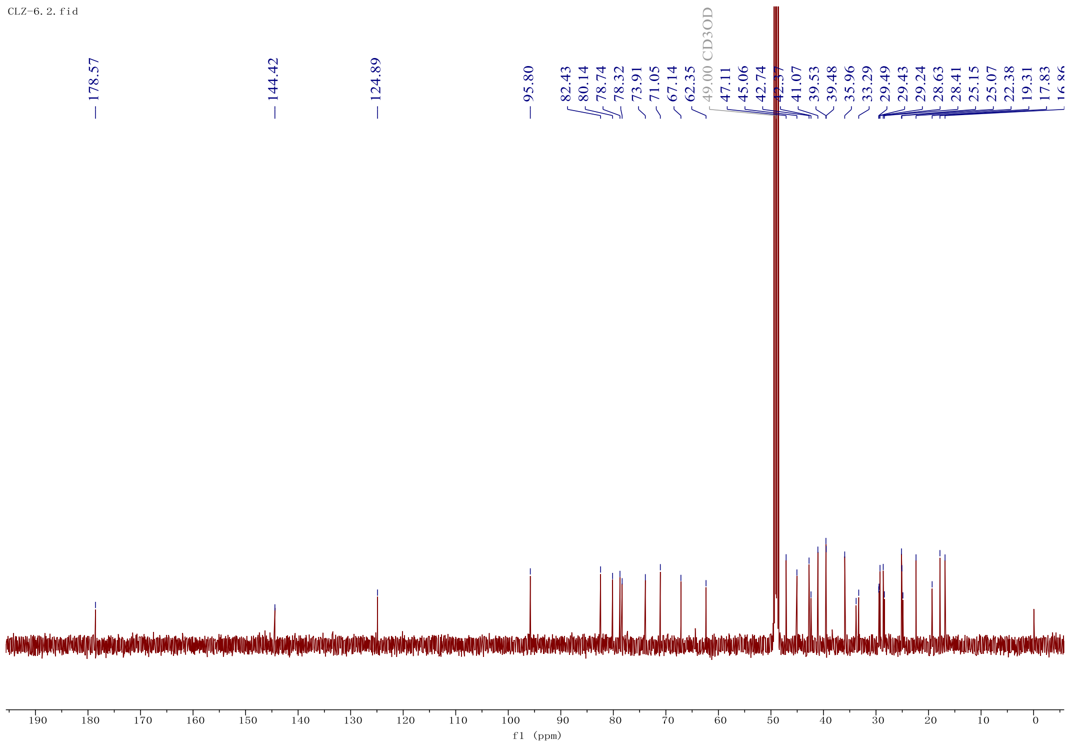

Supplement: Supplementary file 1 [file antioxidants-15-00680-s001.zip › antioxidants-4311038-supplementary.pdf]
